# *Smilodon* sabertooth bite marks, new perspectives on prey-predator models in South American Pleistocene

**DOI:** 10.1186/s12862-026-02551-7

**Published:** 2026-07-07

**Authors:** Yolanda Fernández-Jalvo, M. Dolores Marin-Monfort, Martina Demuro, Gustavo Gómez, Ricardo Bonini, Jonathan Bellinzoni, Sara García-Morato, María A. Gutiérrez, Fernando J. Fernández, M. Teresa Alberdi, Marta Moreno-García, Esperanza Cerdeño, Claudia I. Montalvo, Lee J. Arnold, Pamela Steffan, Jose L. Prado

**Affiliations:** 1 Museo Nacional de Ciencias Naturales (CSIC), José Gutierrez Abascal, 2, Madrid, 28006 Spain; 2 INGEOSUR, CONICET, UNS, Avenida Alem 1253, Bahía Blanca, 8000 Argentina; 3 School of Physics, Chemistry and Earth Sciences, Adelaide University, Room 1 13, Mawson Building, Adelaide City Campus East, Adelaide, 5005 Australia; 4 INCUAPA-CONICET- UNCPBA, Facultad de Ciencias Sociales, Universidad Nacional del Centro de la Provincia de Buenos Aires, Avda. del Valle 5737, Olavarría, 7400 Argentina; 5 Instituto de Historia (CSIC) Albasanz, 26-28, Madrid, 28037 Spain; 6 CONICET-Grupo de Estudios en Arqueometría, Instituto de Química Aplicada a la Ingeniería, Facultad de Ingeniería, Universidad de Buenos Aires (UBA), Av. Paseo Colon 850, Buenos Aires, C1063ACV Argentina; 7 Paleobiología y Paleoecología, Instituto Argentino de Nivología, Glaciología y Ciencias Ambientales (IANIGLA), Centro Científico Tecnológico-CONICET-Mendoza, Avenida Ruiz Leal s/n, Mendoza, M5500 Argentina; 8 Facultad de Ciencias Exactas y Naturales, Universidad Nacional de La Pampa, Uruguay 151, La Pampa, Santa Rosa, 6300 Argentina

**Keywords:** *Smilodon* tooth-mark pattern, Extinction, Megafauna, Human overhunting, Climate

## Abstract

**Background:**

Traditionally, saber-toothed cats, particularly species of the genus *Smilodon*, were considered unable to bite through bones due to the apparent fragility of their teeth. We analyze here megafauna fossil remains of Salto de Piedra palaeontological site (Humid Pampas), one of the few extensively radiometrically dated Pleistocene sites in Argentina and systematically excavated. Salto de Piedra is located near contemporary human occupation sites that have yielded megafauna remains. This spatial and chronological proximity provides an exceptional scenario to compare the prey ranges hunted by humans and by *Smilodon*.

**Results:**

Here we provide *Smilodon populator* tooth mark patterning on their megaherbivore preys. Mark morphology demonstrates that *Smilodon* canines penetrated and processed cortical bone, refuting interpretations that considered them functionally fragile. These tooth marks have been compared with other carnivores and saber-toothed felid fossil sites. This spatial and chronological control provides direct evidence of predatory activity and constrains its temporal span, enabling evaluation of the ecological role of this hypercarnivore.

**Conclusion:**

This extraordinary discovery provides direct evidence of *Smilodon* ‘s predation, and confirms that it was an apex predator, focused almost exclusively on megafauna. These tooth marks can be identified and extrapolated to other fossil sites. Furthermore, this diagnostic tooth-mark pattern enables reconstruction of prey preferences. Our results indicate that *Smilodon* exerted sustained predatory pressure across a broad range of megaherbivores. Despite the spatial proximity and temporal overlap between *Smilodon* predation (22.2–13.3 ka BP) at Salto de Piedra and human arrival to this area (~ 14 ka BP), no interactions between these two predators has been observed indicating certain avoidance between them. The complex predator-prey trophic interactions during the Late Pleistocene–Holocene transition in the Pampas region of South America, support multicausal models, integrating ecological and anthropogenic drivers.

**Supplementary Information:**

The online version contains supplementary material available at https://doi.org/10.1186/s12862-026-02551-7.

## Teaser

Evidence from the iconic saber-toothed cat, *Smilodon*, reveals that megafauna was shared by them and human hunting.

## Significant statement

The Salto de Piedra site, located in the Humid Pampas of Argentina, has yielded an exceptional find: a key contribution, as no study of this kind had ever been carried out on *Smilodon* before. In this paper, we describe and characterise the diagnostic pattern of bite marks attributable to *Smilodon populator* (saber-toothed) on the bones of its prey. Until now, no bite marks on their prey bones had ever been described for this iconic predator, which was thought to have particularly fragile canines for marking bone. Our study shows that *Smilodon* fed on a wide range of megaherbivores, far greater than that found in contemporary human settlements near Salto de Piedra.

## Introduction

Following the end-Cretaceous extinction of non-avian dinosaurs, mammals rapidly diversified, occupying a wide range of ecological niches [1]. Some lineages evolved to attain large body sizes, giving rise to the appearance of the Pleistocene megafauna. In South America, iconic genera such as *Megatherium*,* Glyptodon Notiomastodon*,* Toxodon* or *Macrauchenia*, among others went extinct near the Pleistocene–Holocene boundary [2] approximately 12,900–11,700 years ago (12.9–11.7 ka BP; “kilo-anni–thousand years Before Present”). Researchers have primarily attributed the extinction of these and other megamammals to three drivers: human activity, climate change, or their combined effects [3–5]. The current evidence remains inconclusive as to (a) whether humans were the primary cause for the extinction of the megafauna through overhunting, (b) whether the megafauna was already in decline due to abrupt climate shifts from the glacial (Late Pleistocene) to the interglacial (Holocene) transition [6], (c) or whether the presence of humans accelerated an extinction process initiated by climate change. Climate-based hypotheses emphasize the role of environmental change in reducing habitat suitability for large-bodied taxa, either by decreasing resource availability or intensifying intraspecific competition [7].

**Fig. 1:**
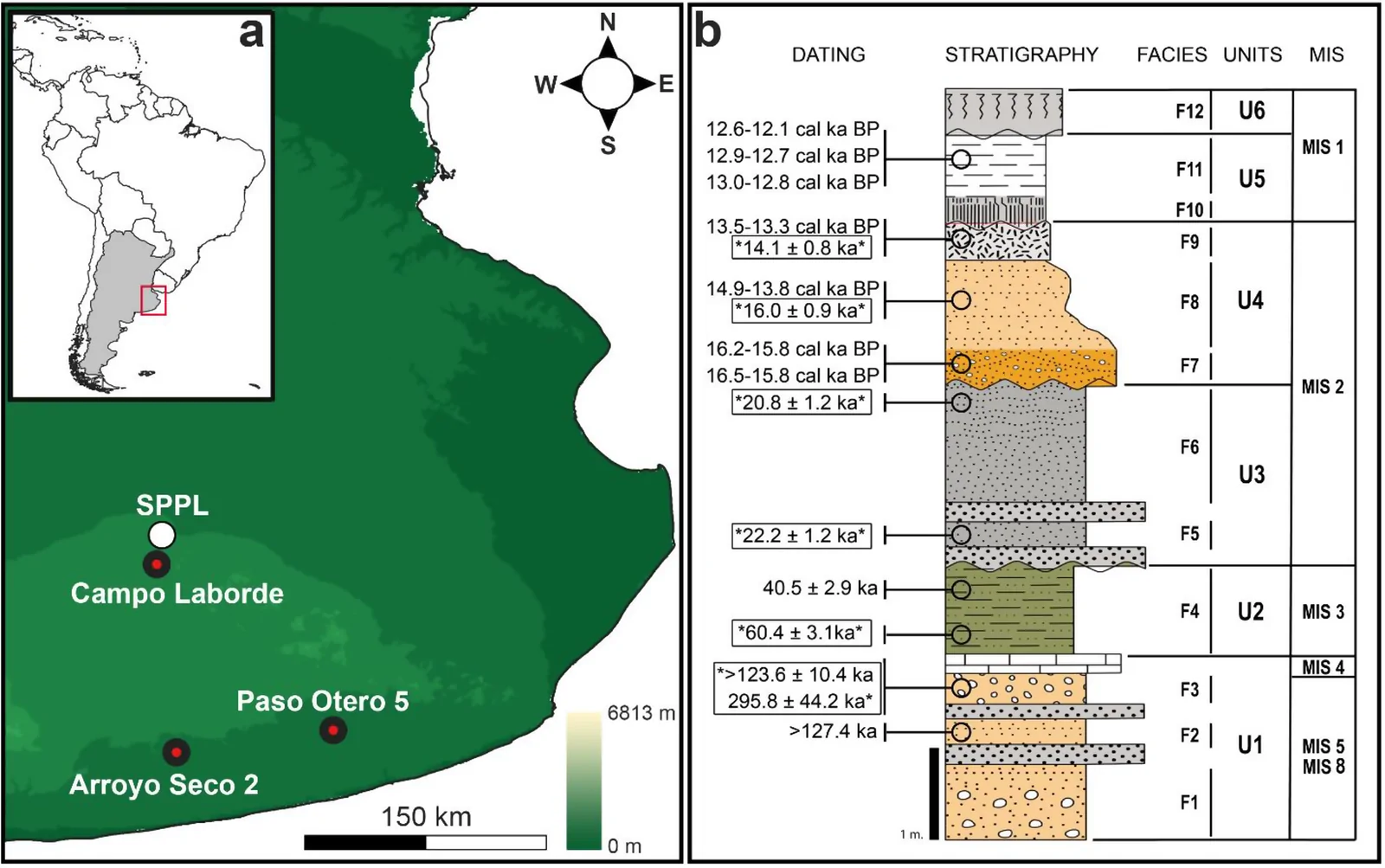
**a**. Map of South America and Humid Pampa with location of SPPL (Salto de Piedra Paleontological Locality, white dot, few kilometers from the town of Olavarria), and contemporary human occupation sites (red-black dots) mentioned in the text are located between 7 km (Campo Laborde) to 180 km (Arroyo Seco 2) or 200 km (Paso Otero 5) from SPPL (see Supplementary Information). **b**. Composite stratigraphic column of SPPL units and radiometric dating. Previously published ages were unboxed [8, 9]. *New dates are boxed (see Supplementary Information). Marine Isotopic Stages (MIS) are climatic and timescale (i.e., even MIS numbers indicate glacial periods, and odd MIS numbers indicate interglacial periods)

Systematic excavations at Salto de Piedra Paleontological Locality (hereafter SPPL; Fig. 1) make it one of the few South American paleontological sites to be both excavated and radiometrically dated [8, 9]. Excavators have recovered a highly diverse megafaunal assemblage [8] with giant terrestrial sloths (*Megatherium*, *Glossotherium*, *Lestodon*, *Scelidotherium*); large cingulates (*Glyptodon*, *Doedicurus*, *Panochthus*, *Neosclerocalyptus*, *Eutatus*); notoungulate (*Toxodon*); and proboscideans (*Notiomastodon*). We have recovered two large carnivores to date from SPPL: the short-faced bear (*Arctotherium bonariense*) from the oldest stratigraphic level (Unit 1, ~ 296 to > 124 ka BP; see Supplementary Information: OSL dating) and *Smilodon populator* from Unit 4, dated to ~ 14.1 ka BP (Fig. 1).

The endemic South American saber-toothed cat, *Smilodon populator*—a machairodontine felid— has the largest body mass of the genus (220–360 kg, exceptionally up to 420 kg), compared to other North American origin relatives, such as *S. gracilis* (comparable to *Panthera onca* or *P. pardus*, between 55 and 100 kg, exceptionally up to 150 kg) and *S. fatalis* (similar size to a Siberian tiger, between 160 and 280 kg, exceptionally up to 340 kg) [10]. *Smilodon* as other megafaunas became extinct around the Pleistocene–Holocene transition [11] ~ 10 ka. Some data suggest that parts of Brazil may have acted as refugia for certain megafaunal species, with isolated populations potentially surviving until ~ 9 ka BP [12]. Upon entering this landscape, early human groups would have encountered other ecological competitors, including *Smilodon*, all targeting equids, camelids, and megaherbivores, such as giant ground sloths. The earliest widely accepted human presence in the Pampas region dates to ~ 14 ka BP [13, 14], although recent studies [15] propose an earlier presence around 20 ka BP, pending further confirmation.

The ecological role of hypercarnivores, particularly *Smilodon*, remains underexplored, and their potential competitive interactions with humans are poorly understood. The paleontological and archaeological record from the intermountain region of the Argentine Humid Pampas provides a unique scenario to compare the predatory pressure exerted by large sized carnivores and humans on megafauna. Notably, SPPL lacks direct or indirect evidence of human activity or artefacts and, therefore, the site exhibits the natural ecological conditions existing in this area when humans arrived at the Pampas. Just seven kilometers from SPPL, Campo Laborde [16], together with other human occupation sites located within 200 km [17], Paso Otero 5, and [18] Arroyo Seco 2 (see Supplementary Information and Fig. 1) — create a broader regional framework for assessing prey preferences of these carnivores and contemporary humans that inhabited the region sharing similar ecosystem.

The characterization of *Smilodon* tooth marks on megafauna bone remains enables the identification of its prey and the reconstruction of its predatory behavior, prey selection and feeding strategies. These data clarify the ecological role of this apex predator and offer new information and new perspectives to predatory models including anthropogenic factors. In this context, the hunting behavior of *Smilodon* may now be considered alongside that of early humans.

## Materials and methods

Additional details are in SI Appendix.

SPPL is located in the upper basin of Tapalqué Creek. This small valley is incised into loessic Plio-Pleistocene sediments, which are discontinuously exposed along the creek banks. The adjacent interfluves are covered by Late Pleistocene and Holocene aeolian deposits that constitute the parent material for most modern soils [19]. The resulting stratigraphic framework consists of six depositional units (U), separated by erosional disconformities and classified as allounits, alongside twelve facies (F) that represent distinct sedimentary sub-environments with variable fossil preservation potential [20]. SPPL site preserves crucial paleoenvironmental evidence pertaining to Late Quaternary climatic evolution within the inter-hill Pampean region. The development of the Perdido–Tapalqué Basin reflects ancient weathering episodes alongside the combined influence of aeolian and fluvial processes operating under variable climatic regimes [21].

Fossils recovered from most Pleistocene sites in the Pampean region of Argentina generally lack detailed and precise stratigraphic resolution, as systematic excavation methods have rarely been applied. In contrast, SPPL stands out as one of the few Pleistocene paleontological sites in Argentina excavated with rigorous archaeological protocols. This systematic approach involved precise recovery of fossils with three-dimensional spatial coordinates using a total station theodolite, resulting in highly accurate stratigraphic context for each specimen within defined depositional units.

We recorded each specimen in situ with a Nikon Nivo 5.C total station, and we labeled the collection bags with a sequential record number and a field reference. We recorded initial three-dimensional stratigraphic data using an arbitrary coordinate system: Y-axis near 1000 m, X-axis near 2000 m, and elevation reference of 150 m. We used a ground control point, located ~ 5 m from Excavation Unit I near the Tapalqué Creek bank, to georeferenced these data in real-world coordinates. We surveyed this point with a DGPS Reach Emlid RS + and post-processed using RINEX observations and RTKLIB software [22]. We then transformed the arbitrary total station data into the Argentine local coordinate system POSGAR 07 (Posiciones Geodésicas Argentinas 2007) using QGIS 3.34 (QGIS Development Team, 2024) and the orthometric height model GEOIDE-AR16.

### Faunal collections of Salto de Piedra site

Specialists conducted comparative anatomical and taxonomic identifications for each mammalian group [8], using reference specimens from the paleontological collections of the Museo de La Plata (MLP) and the Museo Argentino de Ciencias Naturales “Bernardino Rivadavia” (MACN) in Buenos Aires and *Smilodon* collection of Rancho La Brea (Los Angeles, USA). We recovered all specimens discussed in this study during systematic excavations and labelled them with FCS-P followed by correlative numbers. The paleontological collections of the Institute of Archaeological and Paleontological Research of the Quaternary Pampas, National University of the Center of the Province of Buenos Aires (INCUAPA–CONICET–UNICEN) currently house and curate these specimens. In total, we identified 23 genera and 27 species; detailed systematic identifications and the specimens studied for each taxon are listed in Table 2 and in the recently published work [8].

We systematically analyzed surface bone modifications across the entire fossil assemblage from SPPL. The mammalian assemblage includes representatives of Xenarthra (Dasypodidae, Glyptodontidae, Megatheriidae, and Mylodontidae), Litopterna (Macraucheniidae), Notoungulata (Toxodontidae), Proboscidea (Gomphotheriidae), Perissodactyla (Equidae), Artiodactyla (Camelidae, Cervidae), Carnivora (Canidae, Ursidae, Felidae), and Rodentia (Chinchillidae, Caviidae). The assemblage displays the highest abundance of identifiable fossils recovered from U4–F9, which yielded the largest number of identifiable fossils remains, providing detailed occurrences of each taxon across the respective facies and stratigraphic units. This fauna includes typical Late Pleistocene South American mammals, encompassing extinct megafaunal taxa as well as some extant species still present in other regions of the continent.

### Procedure to measure the tooth marks

**Fig. 2:**
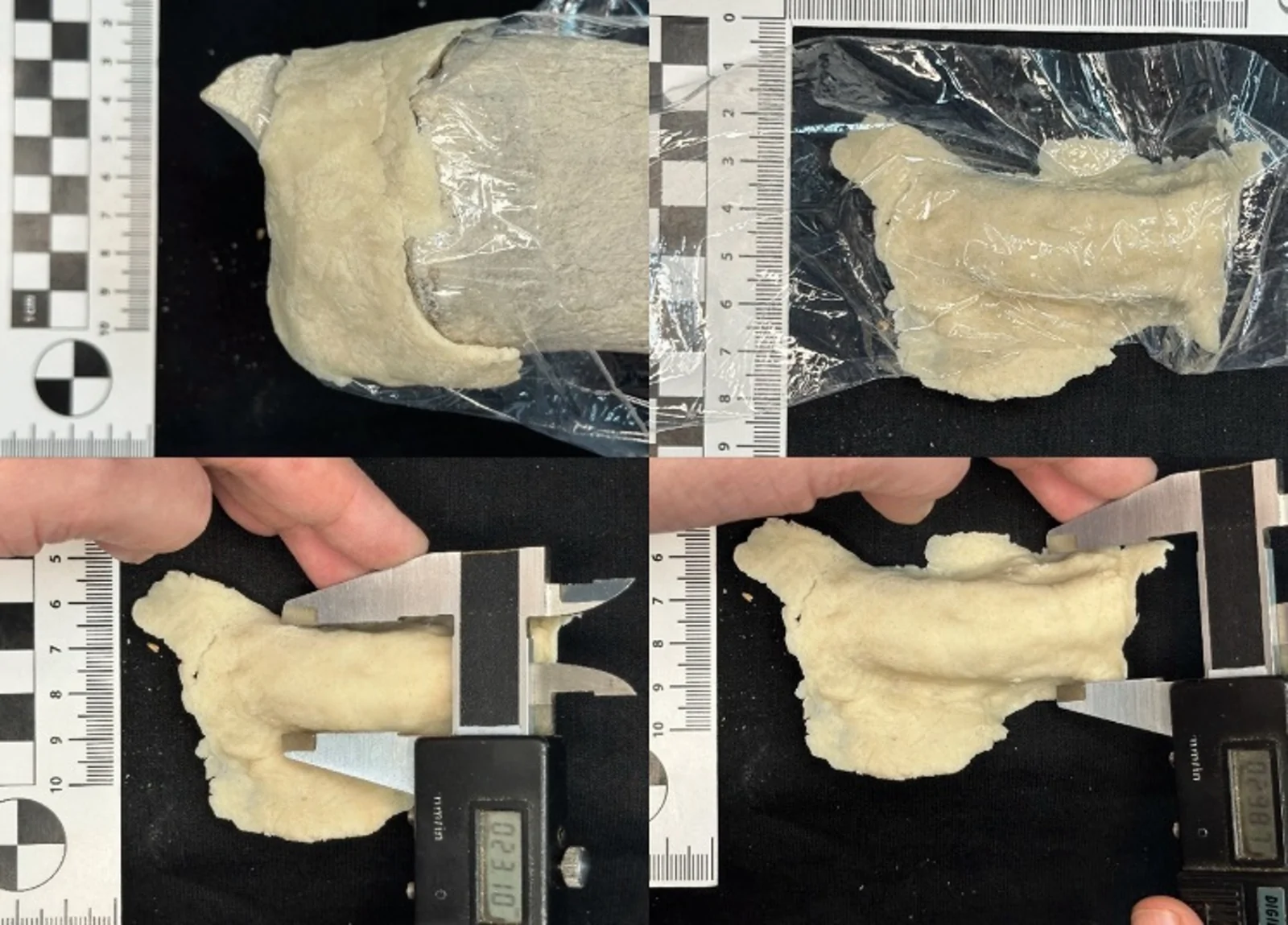
Methods. Measurements taken directly on plasticine molds

We identified and measured two primary types of carnivore tooth marks: punctures and grooves. Punctures are inverted cone-shaped perforations that penetrate the underlying bone tissue, whereas grooves are linear incisions with lengths at least four times their breadth [23]. We took all measurements directly on the bone surface using a digital caliper. When bone morphology or fragility impeded direct access, we used molds made of plasticine for measurement. The bone surface of the fossil was covered with a thin plastic film before being moulded with plasticine to prevent the spongy tissue from sticking to the plasticine (Fig. 2).

For each mark, we recorded three dimensions: length (maximum extension), width (maximum value perpendicular to the length), and depth (maximum value perpendicular to the bone surface). These grooves and the measurements shown in Table 1 reduce the width from one end to the apex, providing two measurements. When recording puncture marks, we excluded peripheral edges caused by dragging or deformation of the bone because these edges do not represent the actual dimensions of the carnivore’s tooth. Additionally, some bones exhibited scooping out, which indicates the complete removal of the epiphysis due to intense carnivorous activity. This modification was recorded descriptively but was excluded from metric analyses due to its extensive and variable nature.

### 3D surface scanning for osteological collections

This section describes the methodology and key considerations for 3D surface scanning of osteological specimens using portable equipment. Surface scanners, such as those using laser or structured light technology, capture the geometry by projecting a light pattern onto the object and recording the reflected signal. The result is a dense 3D point cloud that is later triangulated into a high-resolution polygonal mesh suitable for geometric morphometric analyses [24, 25].

In this study, we used an Artec Space Spider scanner (www.artec3d.com). Scanning involved manually moving the device around the specimen or rotating the specimen on a turntable. Artec Studio v.19 software integrates geometric and texture tracking to maintain scan continuity, with tracking accuracy further improved by placing texture markers or patterned mats beneath the specimen.

The digitization of fossil material has transformed the curation, preservation, and accessibility of osteological collections. High-resolution 3D models enable remote examination, virtual curation, and replication via 3D printing, minimizing the need for direct handling and preserving fragile specimens. In line with the FAIR data principles (Findable, Accessible, Interoperable, and Reusable), all 3D models generated in this study have been uploaded to the open-access repository MorphoSource, with persistent links provided in Table 1. This approach supports transparent, reproducible research and facilitates long-term data reusability. Open access to digitized collections fosters broader participation in scientific inquiry, particularly by institutions and researchers with limited access to physical specimens. Ultimately, digital dissemination not only contributes to the preservation of cultural and biological heritage but also promotes equity, collaboration, and innovation in the global research community.

### Luminescence dating methods

Seven luminescence dating samples were collected from cleaned sedimentary exposures at Salto de Piedra (Olavarría) study sites using PVC tubes and wrapped in light-proof bags for transportation and storage. Bulk sediment samples were also collected from the surrounding few cm of each sample tube for gamma and beta dose rate determination, as well as water content analysis. The two lowermost samples (SDP23-1 and SDP23-2) were collected from unit U1, one sample (SDP23- 3) was collected from U2, two samples (SDP23-6 and SDP23-7) were collected from U3 and the two uppermost samples (SDP23-4 and SDP23-5) were collected from U4 (Fig. 1b).

Environmental dose rates (Table S1) have been determined using sample-specific radionuclide activities of U, Th and U determined from high-resolution gamma-ray spectroscopy (HRGS) measurements (Table S2). HRGS measurements were made on dried and powdered sediment samples collected immediately adjacent to the luminescence dating samples using a p-type, high-purity, germanium well detector. HRGS data analysis approaches are described in the Supplementary Information. External beta and gamma dose rates were then calculated from the HRGS radionuclide activities using the conversion factors [26]. Cosmic doe rates and internal dose rates were also included as described in the Supplementary Information (main text and footnotes in Table S1).

Three semi-independent luminescence dating signals were used to obtain a chronology for Salto de Piedra. Single-grain optically stimulated luminescence (OSL) of quartz was applied to upper samples from U3 – U4 and sample SDP23-2 from U1, whilst single-grain thermally-transferred OSL (TT-OSL) dating of quartz and multi-grains post infrared-infrared stimulated (pIR-IRSL) luminescence dating of K-rich feldspar grains was routinely applied to the two lower samples collected from U1. TT-OSL and pIR-IRSL are extended-range luminescence dating approaches typically preferred over OSL when dating older samples due to the higher dose saturation properties of these signals [27, 28] (e.g., Middle Pleistocene; >150 ka). The use of these extended-range luminescence signals was deemed necessary for the U1 samples due to the high accumulated dose of > 250 Gy measured for sample SDP23-2 (U1) using single-grain OSL, which indicated that U1 was > 150 ka (Table S8).

Luminescence dating was conducted at the University of Adelaide’s Prescott Environmental Luminescence Laboratory. For single-grain OSL and TT-OSL measurements, quartz grains of various size ranges (90–125 μm; 180–250 μm; 212–250 μm) were collected and measured for each sample depending on their abundance (see Table S1). For pIR-IRSL measurements, K-feldspar grains (90–125 μm diameter) were extracted. The un-illuminated centres of the tubes were extracted under safe light (dim red LED) conditions and prepared using standard procedures [29]. Silicate grains were then etched by hydrofluoric (HF) acid to remove the alpha-irradiated external layers (10% HF digestion for 10 min to etch the K-feldspar fractions; 48% HF digestion for 40 min to etch the quartz fractions. The etched grains were then rinsed in 30% hydrochloric acid to remove any precipitated fluorides and re-sieved using a 63 μm sieve to eliminate any disaggregated grains.

Quartz OSL and TT-OSL and K-feldspar pIR-IRSL D_e_ measurements were made using the instrumentation, single-aliquot regenerative-dose (SAR) procedures and quality assurance criteria [30, 31]. Single-grain OSL, single-grain TT-OSL and multi-grain pIR-IRSL dose recovery tests were performed on samples SDP23-6, SDP23-1 and SDP23-2, respectively, to determine the suitability of the SAR protocols outlined in Table S3. The single-grain OSL quartz SAR protocol includes a preheat of 240 °C for 10 s and 200 °C for 10 s prior to measurement of the natural (L_n_), regenerative dose (L_x_) and test-dose (T_n_ and T_x_) OSL signals, respectively (Table S3). These preheating conditions yielded an accurate measured-to-given dose ratio of 1.00 ± 0.04, and an overdispersion value of 2 ± 1%, for a ~ 55 Gy dose recovery test performed on individual grains of SDP23-6. The single-grain TT-OSL produced a dose recovery ratio of 1.10 ± 0.10 in agreement with unity (Table S5). For the pIR-IRSL signal, the elevated temperature made at 225 °C (pIR-IRSL_225_ protocol) produced a dose recovery ratio of 0.97 ± 0.03, which is in agreement with unity at 1σ, while the elevated temperature post-IR measurements made at 290 °C (pIR-IRSL_290_ protocol) resulted in dose overestimation at 2σ (dose recovery ratio = 1.13 ± 0.03). We have hence opted to use the pIR-IRSL_225_ protocol to date the K-feldspar fractions from U1 at Salto de Piedra (Table S6).

Single-grain OSL D_e_ measurements were made on 200–5100 individual quartz grains per sample (Table S4), single-grain TT-OSL measurements were made on 800–2400 individual quartz grains per sample (Table S4) and multi-grain pIR-IRSL D_e_ measurements were made on 12–16 aliquots of K-feldspars per sample (each aliquot containing ~ 360 grains). The natural D_e_ datasets exhibit low to moderate overdispersion values (1–47% for the single-grain OSL and TT-OSL datasets; 20–32% for the pIR-IRSL datasets; Tables S8–S10) and are generally not considered to be substantially skewed according to the weighted skewness test [32, 33]. These D_e_ distribution characteristics are consistent with those commonly reported for well-bleached, unmixed samples [32], hence we have used the weighted mean D_e_ values (calculated using the central age model [34] to derive final burial dose estimates.

Anomalous fading tests were performed on K-feldspar aliquots that had been measured for D_e_ determination, following published procedures (see Supplementary Information). The results of these anomalous fading tests are presented in Table S7 and show fading rates values of ~ 1.90%/decade for sample SDP23-2 and ~ 3.5%/decade for sample SDP23-1. The average g-value for these samples (2.56 ± 0.46) is higher than the 1–2%/decade range commonly reported for pIR-IRSL_225_ and pIR-IRSL_290_ datasets that are considered athermally stable [35]. Therefore, we have applied an empirical fading correction to the pIR-IRSL_225_ ages obtained for samples SDP23-1 and SDP23-2 using the measured *g*-values [36].

## Results

### *Smilodon* tooth marks on prey bones (new discovery)

**Fig. 3:**
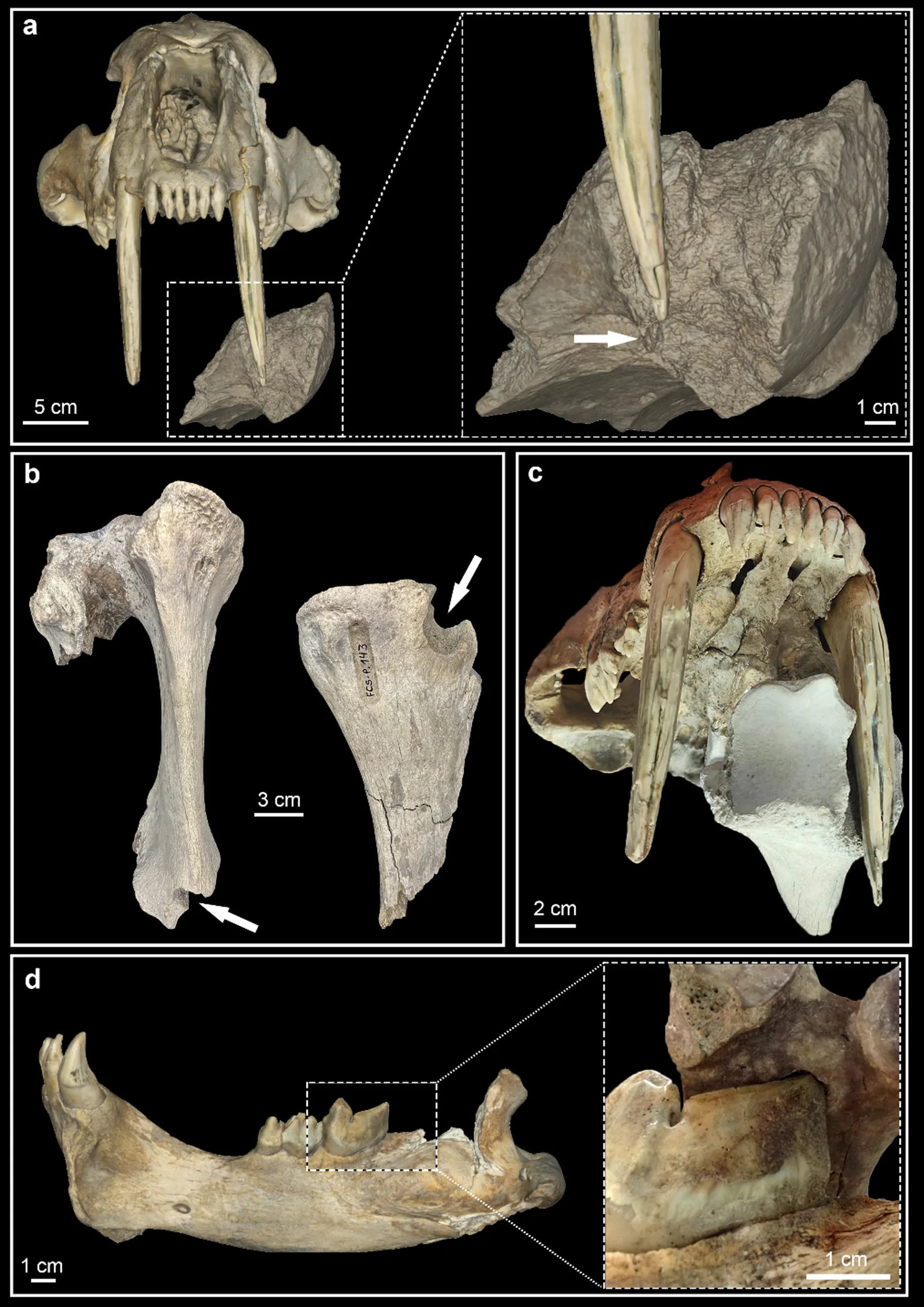
*Smilodon* tooth marks. **a**: *Smilodon* skull superimposed on a head of humerus (FCS-P.156). Right: detail of the bite mark and the upper canine of *Smilodon* matching in size and shape (white arrow). **b**: *Toxodon* tibiae with large and deep conical grooves (white arrows): left, FCS-P.25 distal end; right: FCS-P.143 proximal end. **c**: *Smilodon* skull piercing the *Toxodon* tibia FCS-P.25. **d**: Adult *Smilodon* mandible. The small box frames both the rib and the carnassial tooth of *Smilodon* that has penetrated it

The first specimen displaying a clear shape of a canine impression is the proximal end of a humerus of *Toxodon platensis* (Fig. 3a). Several megafaunal skeletal elements exhibit similar unusually large, wide, conical grooves (i.e., width reduction along their length)— a pattern that has never been described in the literature before — consistent with *Smilodon* canine morphology (Fig. 3a and c). These tooth marks contradict previous hypotheses suggesting that *Smilodon* avoided contact with bones while feeding to prevent the slightest risk of fracturing their oversized canines [37, 38]. These characteristic marks offer new insights into the feeding behavior of *Smilodon*. In fact, durophagy was already indicated by dental microwear analysis [39] from Rancho La Brea (Los Angeles, USA) and injuries on *Smilodon populator* skulls [40].

Two *Toxodon* tibia-fibula display well-defined, deep, conical grooves (Fig. 3b), closely matching the dimensions of *Smilodon* canines (Fig. 3c). *Smilodon* pierced these bones, leaving grooves that range from shallow (15–8 mm wide; Fig. 3a) to deeply impressed (21–33 mm wide; Fig. 3c, Table 1), that exhibit a diagnostic conical shape, a pattern attributable to *Smilodon* canine morphology. Two megaherbivore long bones—a femur and a humerus—show a distinct type of carnivore-induced damage known as “scooping out,” a modification also identified in extant large felids and other saber-toothed cat’ feeding behavior [41–43].

**Fig. 4:**
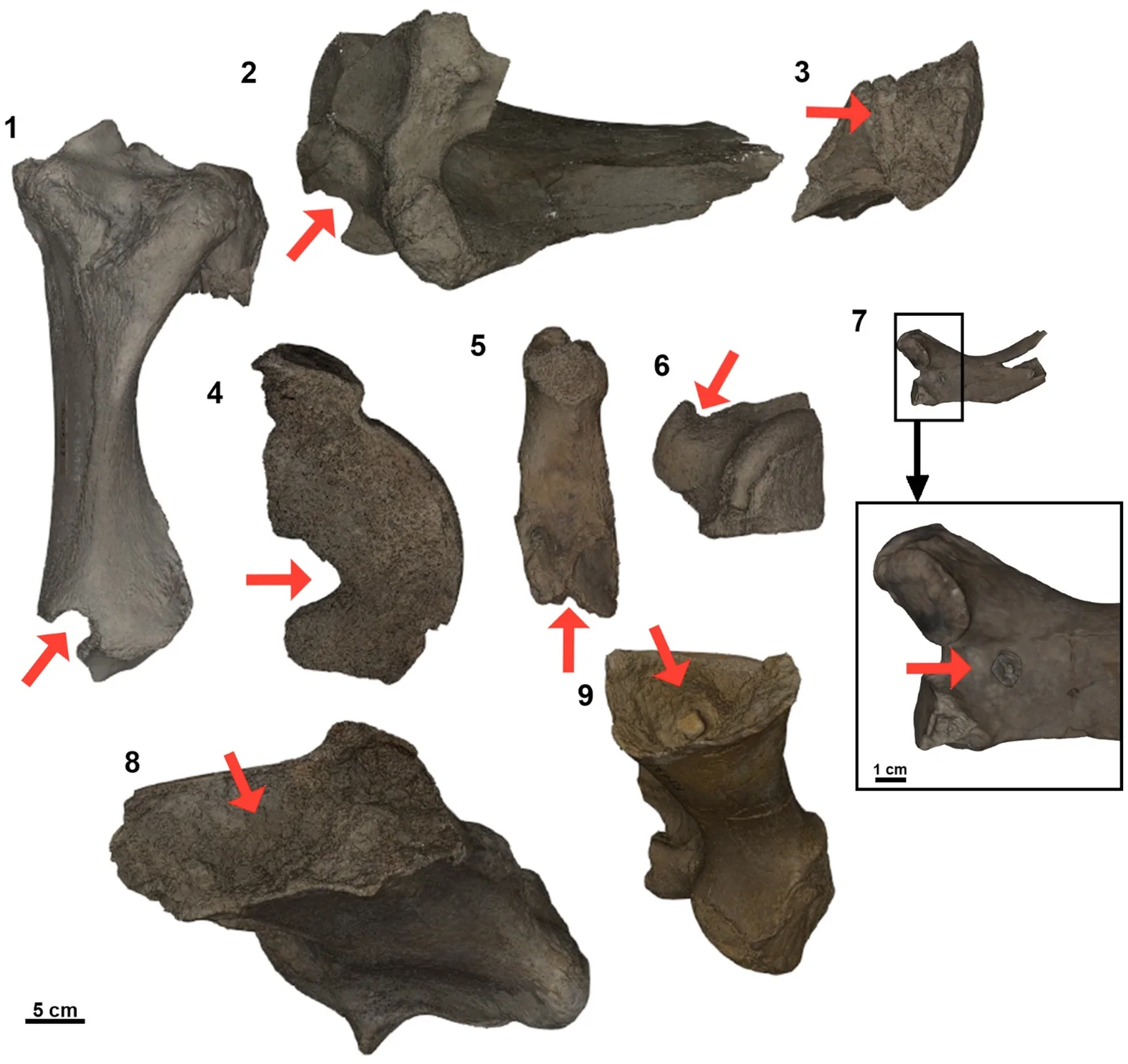
3D surface scanned images. **4.1**. FCS-P.25 – Tibio-fibula of *Toxodon platensis* –with distal deep conical groove. **4.2** FCS-P.143 –Tibio-fibula of *Toxodon platensis* –with proximal deep conical groove. **4.3** FCS-P.156– Humerus of *Toxodon platensis* –with shallow conical groove. **4.4.** FCS-P.17 Humerus of *Lestodon armatus*. **4.5.** FCS-P.145– Metacarpal of *Lestodon armatus* –with shallow conical groove. **4.6.** FCS-P.13 – Astragalus of *Doedicurus clavicaudatus* –with shallow conical groove. **4.7.** FCS-P.242–1st rib of megafauna –with carnassial puncture marks **4.8**. FCS-P.160 – Femur of *Glossotherium robustum* –with scooping out. **4.9**. FCS-P.303 – Humerus of *Notiomastodon platensis* –with scooping out. (3D image links are displayed in Table 1)

Characteristic bite marks attributed to *Smilodon* have been identified in fossils from Units 3 and 4 (dated between 22.2 and 13.3 cal kyr BP). These modifications (Fig. 4) described in SPPL megafauna provide direct evidence of carcass processing by *Smilodon*, in the same way that human-induced cut marks are interpreted as indicators of human butchery. Note that not all bones of the prey skeleton consumed by carnivores bear tooth marks [44–46], just as cut marks are not present on all bones of slaughtered animals [16–18]. We can assume that these marks were not intentionally made, but rather the result of accidental contact between bone and the carefully shaped edge of a stone tool or potentially breakable teeth [23].

**Fig. 5:**
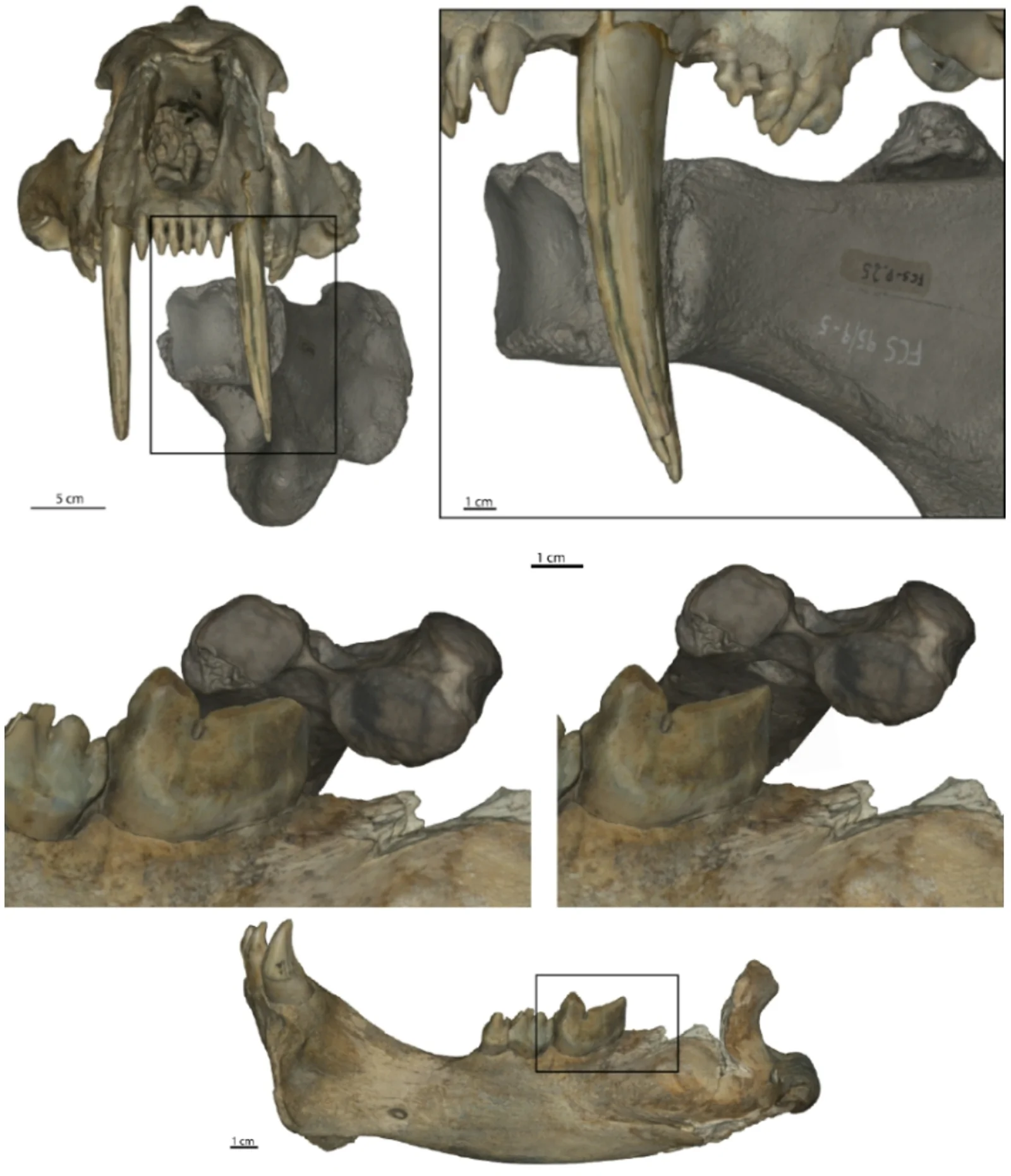
3D scanned figures of skull and mandible of *Smilodon populator* superimposed to fossils from Salto de Piedra Paleontological Locality (SPPL) Top: *Smilodon* skull superimposed to FCS-P.25 showing the penetration of the saber-toothed canine into the distal tibia of *Toxodon platensis* leaving a deep conical large groove (see the 3D images). Bottom: Puncture marks match with the lower and upper carnassial teeth penetrating into the rib (FCS-P.242) with the most prominent cusps of *Smilodon*, as described in the Main text. *Skull and mandible MHM-P 102 (associated) of *Smilodon populator* (Stratigraphic provenance: Lujan Formation, Late Pleistocene, ca. 14 ka, Lujanian Stage/Age). Geographical location: near La Chumbiada, G. Belgrano; housed in the “Alfredo E. Múlgura” Municipal History Museum in General Belgrano, Buenos Aires

A large rib fragment displays a special case (FCS-P.242) from SPPL showing bilateral puncture marks consistent with *Smilodon* ’s carnassial dentition. One puncture at the vertebral end aligns with the protoconid of the lower carnassial (Fig. 3d), while a shallower, opposing mark matches the paracone of the upper carnassial, indicating the rib was compressed between both jaws (see Table 1; Fig. 5).

**Table 1:** *Smilodon* tooth marks and measurements (mm)

Label FCS-*P*. (Unit/Facies)	Anatomical element	Taxonomic identification	Damage	Length	Width	Depth	3D scan image
25 (U3/F6) (Fig. 4.1)	Tibia-fibula		Canine conical groove	64.01	29.87 21.63	14.58	www.morphosource.org/concern/media/000756679
143 (U3 F6) (Fig. 4.2)	Tibia-fibula		Canine conical groove	48.67	43.66 33.47	26.69	www.morphosource.org/concern/media/000756691
156 (U3/F6) (Fig. 4.3)	Humerus		Shallow canine conical groove	67.96	13.14 7.8	7.72	www.morphosource.org/concern/media/000756695
			Carnassial tooth mark on crenulated fracture	6.41			
17 (U4/F9) (Fig. 4.4)	Humerus		Canine groove	60	49 − 24	40	www.morphosource.org/concern/media/000756654
145 (U3 F6) (Fig. 4.5)	Metacarpal		Shallow canine groove	45	15	10	www.morphosource.org/concern/media/000756671
13 (U4/F7/8) (Fig. 4.6)	Astragalus		Shallow canine conical groove	29.94	15.07 13.03	8.20	www.morphosource.org/concern/media/000756708
242 (U4/F9) (Fig. 4.7)	1st Rib	indet	Lower carnassial puncture	6.97	5.38	2.76	www.morphosource.org/concern/media/000756721
			Upper carnassial shallow puncture	3.62	2.28	1.81	
160 (U4/F7/8) (Fig. 4.8)	Femur		**Scooping out** consists of complete removal of the articular ends and part of the shaft cancellous tissue eaten away, leaving thin walls of the cortical bone and part of the trabecular bone at the bottom of the cavity [44].				www.morphosource.org/concern/media/000756734
303 (U4/F7/8) (Fig. 4.9)	Humerus						www.morphosource.org/concern/media/000756726

The width of the conical grooves decreases along their length. Two measurements are shown: one taken at one end and the other taken at the narrowest point. Anatomical elements have been 3D scanned and links are displayed in the right-hand column

Carnivore related damage is the taphonomic modification that affect all these fossil bones referred in Table 1, weathering affects 50% of these fossils at a low or very low rate, abrasion has not been detected in these fossils and disperse oxide stains of iron and manganese affect 50% of these fossils. All these taphonomic modifications are related to the humid ambient of a fluvio-lacustrine environment of deposit.

## Discussion

### The origin of the SPPL fossil association

SPPL corresponds to fluvial, floodplain, and lacustrine-palustrine environments ranging from the Late Pleistocene to the Holocene [20]. The taphonomic modifications observed in these large fossils studied here are consistent with these environments. More specifically, the absence of abrasion indicates that these fossils were not transported. Therefore, the most likely situation is that bones of these animals were buried where they were killed. This suggests that the SPPL fossil association represents an ancient biocenosis, or paleocommunity. These water-associated settings attracted a wide range of wildlife and likely served as ambush sites for predators targeting animals approaching the water’s edge to drink. Predators take advantage of this opportunity to hunt animals that find it more difficult to escape while drinking.

### The role of *Smilodon* vs. other carnivores

A key feature of the SPPL assemblage is the presence of discrete, isolated, and well-defined tooth impressions—produced by both canines and carnassials—on nearly all modified bones. This pattern aligns with felid feeding behavior [44–46] and contrasts markedly with the extensive gnawing and overlapping marks typically produced by canids [45] and ursids [44, 46]. For example, intense gnawing observed on a *Toxodon platensis* humerus head (see www.morphosource.org/concern/media/000756695) is likely attributable to secondary scavenging following *Smilodon* abandonment of the carcass. The medium-sized canid *Dusicyon avus* (~ 16 kg) [47], recorded in Unit 4, produced small marks on skeletal elements of *Dolichotis patagonum* (mara, a large rodent) and may have contributed to the modification of the *Toxodon* humerus head.

Each carnivore taxon leaves characteristic modifications based on its dentition and feeding behavior. The short-faced bear *Arctotherium bonariense* (~ 370 kg) [45], present in Unit 1 (> 124 ka) tends to scavenge and leave multiple tooth marks instead of isolated marks, as other ursids [44, 46]. Other large felids known from Late Pleistocene Pampean localities [48, 49], such as jaguars (*Panthera onca*, < 190 kg)] and pumas (*Puma concolor*, 40–80 kg) lack hypertrophied canines, have smaller carnassials, which cause smaller tooth marks on bones [49], and show no evidence at SPPL—neither fossils nor tooth marks.

**Table 2:** Taxonomic list of the mammalian assemblage identified at the SPPL site

UNIT	U1			U2	U3		U4			U5		U6	Stage/Age ^(25)^
Facies	F1	F2	F3	F4	F5	F6	F7	F8	F9	F10	F11	F12	
*Eutatus seguini*		X											BO (FAD) – LU
*Glyptodon munizi*	X												EN (EXCL.)
*Glyptodon reticulatus*						X	X	X	X				BO (FAD) – LU
*Doedicurus clavicaudatus*						X	X	X	X				LU (EXCL.)
*Neosclerocalyptus paskoensis*							X		X				LU (EXCL.)
*Panochthus* sp.						X			X				EN (FAD) – LU
*Megatherium americanum*		X				X							BO (FAD) – LU
*Glossotherium robustum*							X	X					BO (FAD) – LU
*Lestodon armatus*							X	X	X				BO (FAD) – LU
*Scelidotherium leptocephalum*						X							BO (FAD) – LU
*Toxodon platensis*						X	X		X				BO (FAD) – LU
*Macrauchenia patachonica*									X				BO (FAD) – LU
*Dolichotis patagonum*									X				LU (FAD) – Act.
*Lagostomus maximus*									X				PL
*Arctotherium bonariense*		X											BO (FAD) – LU
*Smilodon populator*									X				EN (FAD) – LU
*Dusicyon avus*									X				LU (FAD) – PL
*Hippidion principale*		X							X				EN (FAD) – LU
*Hippidion devillei*						X			X				MA (FAD) – LU
*Equus neogeus*	X	X				X			X				LU (EXCL.)
*Notiomatodon platensis*						X	X	X					BO (FAD) – LU
*Palaeolama* sp.		X											EN (FAD) – LU
*Lama guanicoe*		X				X			X				MA (FAD) – Act.
*Hemiauchenia paradoxa*		X							X		X		MA (FAD) – LU
*Hippocamelus sulcatus*							X	X					BO (FAD) – LU

The table includes all genera and species determined from the systematic excavations at SPPL, specifying their occurrence across the defined depositional units (U) and sedimentary facies (F). Ensenadan (EN), Bonarian (BO), Lujanian (LU), Platan (PL), Actual (Act.), First Appearance Datum (FAD), Exclusive (EXCL)

The most parsimonious explanation is that *Smilodon populator* was primarily responsible for the bone modifications at SPPL on *Toxodon*, *Glossotherium*, *Lestodon*, *Doedicurus*, and *Notiomastodon*. These modifications indicate that *Smilodon* was capable of processing even dense cortical bone over a time span of more than 7,500 years, which suggests a consistent behavior for *Smilodon*. Against previous hypotheses that *Smilodon* could not even touch bone [37, 38], we have found that dense bones of megafauna bear imprints of *Smilodon*’s upper canines without suffering any consequences (Fig. 3a, b and c), that is, break the canines when feeding or, at least, no canine fragments have been recovered from SPPL sediments. Perhaps the pierced herbivore’s bones were weakened or were damaged in fragile parts or had some pathology affecting its consistency. Nonetheless, our current taphonomic and histotaphonomic investigations in progress do not support any bone weakness due to pathological, traumatological or *ante mortem* origin. Most likely, the canines in living animals have the irrigation supply and moisture making the bioenamel itself much more resistant than expected.

The bilateral carnassial punctures found in the FCS-P.242 and the conical grooves found on the long bones of megafauna (Table 1) were recovered from Unit 3-facies 6 to Unit 4-facies 9—the richest layers in herbivore remains (Table 2). Furthermore, the diversity of the faunal assemblage and the absence of taphonomic signatures of environmental stress in this unit lasting more than 7,500 years do not supports a scavenging action. All this suggests that bone chewing behavior was not ecologically compelled, but rather facultative nutritional behavior.

### Tooth mark patterns: *Smilodon* vs. other saber-toothed cats

The tooth mark assemblage at SPPL attributed to *Smilodon populator* includes deep punctures and furrowing consistent with the use of both hypertrophied canines and robust carnassials. This pattern contrasts with that of North American Homotherini saber-toothed cats (Machairodontini), such as *Xenosmilus hodsonae* from Haile 21 A (Florida) and *Homotherium serum* from Friesenhahn Cave (Texas), which produced diagnostic incisor (front teeth) marks on prey bones, particularly on peccary and proboscidean remains [41, 50]. Incisor furrowing—a hallmark of Homotherini carcass processing—, is notably absent at SPPL. Instead, repeated use of large canines and carnassials defines a distinctive “*Smilodon* -type groove” or “SPPL signature”.

Behavioral differences reinforce this distinction. Homotherini sites often show evidence of denning behavior, with dense bone accumulations suggesting repeated use of specific locations for carcass consumption [41, 50]. In contrast, SPPL represents an open-air locality with no evidence of dens or spatially clustered remains. The distribution and condition of bones imply that *Smilodon* consumed carcasses near kill sites, potentially reflecting a more mobile, ambush-based predation strategy.

The absence of incisor marks at SPPL likely reflects both morphological and functional differences between the lineages. In *Smilodon*, the incisors are relatively gracile and laterally displaced to accommodate hypertrophied canines, limiting their role in dismemberment. Studies have documented scooping and furrowing behaviors in both *Xenosmilus* [41] and *Smilodon* (Table 1; Fig. 4.8*Glossotherium* femur and 3.9 *Notiomastodon* humerus) and indicate active bone processing behavior rarely attributed to *Smilodon* [10].

### Human–*Smilodon* interactions nearby SPPL

The proximity of SPPL to several Late Pleistocene archaeological sites (Fig. 1a) offers an exceptional framework to explore potential ecological interactions between apex predators and early hunter-gatherers. Campo Laborde, located ~ 7 km from SPPL, preserves lithic artifacts in direct association with a nearly complete *Megatherium americanum* skeleton bearing cut marks and impact fractures attributed to human activity [16] (Table 3). Similar archaeological-rich contexts occur at Arroyo Seco 2 [17] and Paso Otero 5 [13, 18].

Previous studies documented clear evidence of human modification on *Megatherium americanum* bones, equids, guanacos, and *Hemiauchenia*, but not for other megafaunal taxa, each represented by only 1 or 2 individuals (Table 3). Isolated fragmented bones, representing a single megamammal individual at the human occupation sites —some thermally altered and interpreted as fuel (e.g., Paso Otero 5) [18]— suggest opportunistic use (bone gathering) rather than systematic megafaunal prey exploitation. In contrast, the range of bones modified by *Smilodon* at SPPL encompasses a broader spectrum of megaherbivores bearing evidence of consumption (Table 3), indicating a stronger and more consistent predatory pressure exerted by *Smilodon* than by contemporary humans.

**Table 3:** Megafauna fossils recorded in human and non-human sites

	Salto de Piedra (U3 + U4)	Campo Laborde	Paso Otero 5	Arroyo Seco 2	Average body size (kg)
*Notiomastodon platensis*	X*				4311
*Megatherium americanum*	X	X**	X	X**	2543
*Mylodon* sp.			X	X	2000
*Lestodon armatus*	X*		X		1918
Lestodontinae cf. *Lestodon*				X	1918
*Toxodon platensis*	X*			X	1187
*Toxodon* sp.			X		1187
*Glossotherium robustum*	X*			X	1041
*Glossotherium* sp.			X		1041
*Doedicurus clavicaudatus*	X*				708
*Doedicurus* sp.		X			708
*Panochthus* sp.	X				701
*Scelidotherium leptocephalum*	X				633
*Scelidotherium* sp.			X		633
*Macrauchenia patachonica*	X		X		530
*Macrauchenia* sp.				X	530
*Hippidion principale*	X				476
*Hippidion* sp.				X	476
*Glyptodon reticulatus*	X				457
*Glyptodon* sp.	X		X	X	457
*Equus neogeus*	X		X	X**	380
*Neosclerocalyptus* sp.	X	X			360
*Smilodon populator*	X				328
**Total taxa recorded**	**15**	**3**	**9**	**9**	
**Consumed taxa**	5*	1**	Hemiauchenia**	2**	

Megaherbivores (above 1,000 kg) and large sized mammals (between 999–360 kg) [51, 52]. Asterisks indicate consumption modifications: *tooth marks by *Smilodon* or **human induced modifications, at the sites mentioned in the text. Paso Otero 5 shows possible human induced modifications only in bones of *Hemiauchenia* (extinct camelid with average weight ~ 300 kg). Only *Megatherium americanum* and a few large mammal (*Equus neogeus*, *Hemiauchenia* sp.) present evidence of human processing and consumption [53]

It has been proposed that large carnivores in southern Patagonia became extinct before megafauna due to early human presence [54]. However, more recent analyses based on an expanded radiometric dataset suggest no such temporal offset, placing megafaunal extinction at approximately 12.3 ka BP, coincident with abrupt aridity, environmental changes, and human occupation [4]. These processes could have triggered cascading extinctions as suggested by “Second Order Predation Theory” [55], which posits that humans reduced predator numbers, allowing megaherbivore populations to expand unsustainably, causing environmental degradation and further extinctions.

There is no evidence at the sites in the Pampean Region considered here to suggest any interaction between humans and *Smilodon.* No human remains, artefact or trace of human modifications have been recovered from SPPL. Likewise, the complete absence of *Smilodon* remains at the archaeological sites —Campo Laborde, Arroyo Seco 2 or Paso Otero 5— despite the potential symbolic or utilitarian value of their canines contrasts with documented use of carnivore bones and teeth by humans in other continents [56]. This spatial separation supports a scenario of mutual avoidance.

Most of South America’s megafauna became extinct around the beginning of the Holocene, a pattern that was repeated throughout the world, outside Africa [2, 57]. In the Pampas, human arrival during the Antarctic Cold Reversal approximately 14,000 years ago [6, 14] was followed by a broader demographic expansion during the Younger Dryas (12,900–11,700 years ago). These humans brought with them fluted projectile points optimized for hunting megafauna [2]. At the time when the megafauna population declined to extinction, an extreme drought, loss of tree cover and high aridity climate change affected the southern hemisphere [11, 58, 59], followed by an increase in humidity and temperature during the Holocene [19, 60]. The arrival of humans coincided with this global climate change, and it is possible that their hunting activity contributed to the decline of megafauna and ecological stress. Some authors also propose an unprecedented fire activity recorded at Rancho La Brea apparently triggered by humans [61] caused or, at least, accelerated the ecosystem change where megafauna inhabited. Nonetheless, there has been no record of increased fire activity in the Pampas, whether related to humans or not.

## Final remarks

The fossil remains of megafauna at the Salto de Piedra site in the Argentine Pampas show bite marks that match the size and shape of *Smilodon* teeth. Based on the characteristic tooth marks present on the fossil bones, its prey item on which this hypercarnivore was feeding can be identified. This extraordinary finding provides direct evidence of *Smilodon* ‘s predation confirming that *Smilodon* was an apex predator, focused almost exclusively on megafauna.

This discovery is particularly interesting in relation to the Pleistocene-Holocene transition in the region of the Humid Pampa fossil sites considered in this paper. Dating results obtained in SPPL compared with those human occupation sites (Campo Laborde [16], Arroyo Seco 2 [17] and Paso Otero 5 [18]) shows that signs of *Smilodon* predation exerted a more intense pressure on megafauna than humans did. There is also no evidence that humans targeted *Smilodon*, displaced it from territories, or limited its food resources. In fact, *Smilodon* and humans, despite the proximity between SPPL and the human occupation sites show mutual avoidance. Climatic and ecological contexts of that time and area provides further insight supporting the idea that climate-driven habitat loss and trophic collapse were the important factors leading to the extinction of highly specialized feeders and predator [62, 63]. *Smilodon* most likely lacked the ecological flexibility to adapt to rapid environmental change, concurrent loss of prey, and critical habitats [10]. Instead, early humans adapted and fed on animals that still exist today, such as guanacos, deer, greater Rhea, maras, and hares [53].

The anthropological sites in the Humid Pampa considered here (Table 3) show small and nomadic human populations [14, 53]. Even several thousand years later, the indigenous populations encountered by Europeans maintained lifestyles similar to those of the first nomadic hunter-gatherers settlers. However, during the Pleistocene-Holocene transition, humans increased the pressure on a highly specialized megafauna that were already been affected by drastic climate changes from the Pleistocene glacial period to the Holocene interglacial. In contrast, a resilient fauna of large mammals (smaller in size and more adaptive) persisted, much of which remains unthreatened even today, on which other carnivores of much smaller body mass than *Smilodon* (e.g., *Panthera onca* or *Puma concolor* [64] adapted to the new Holocene environments and climates.

Taken together all this evidence, a multicausal extinction model of the late Pleistocene megafauna, at least in the Southern Cone of America, result of synergistic interaction of multiple ecological pressures [3–6, 11]. This pattern underscores the complexity of extinction dynamics, where climate, ecological specialization, new predators, and interspecific competition converged to drive faunal turnover. Thus, single-cause explanations are inadequate for understanding Late Pleistocene biodiversity loss.

## Supplementary Information

Below is the link to the electronic supplementary material.

Supplementary Material 1

## Data Availability

All data needed to evaluate the conclusions in the paper are present in the paper and/or the Supplementary Materials.
