# Supplementary material for: Smilodon sabertooth bite marks, new perspectives on prey-predator models in South American Pleistocene
Source: BMC Ecol Evol. 2026 Jul 7;26:77. doi: 10.1186/s12862-026-02551-7 (PMC13548479; doi:10.1186/s12862-026-02551-7)
Supplement: Supplementary file 1 [file 12862_2026_2551_MOESM1_ESM.docx]

**Supporting Information for**

*Smilodon sabertooth bite marks, new perspectives on prey-predator models in South American Pleistocene*

Yolanda Fernández-Jalvo^1^ *, M. Dolores Marin-Monfort ^1,2^, Martina Demuro ^3^, Gustavo Gómez ^4^, Ricardo Bonini ^4^, Jonathan Bellinzoni ^4^, Sara García-Morato ^1,5^, María A. Gutiérrez ^4^, Fernando J. Fernández ^6^, M. Teresa Alberdi ^1^, Marta Moreno-García ^5^, Esperanza Cerdeño^7^, Claudia I. Montalvo ^8^, Lee Arnold ^3^,

Pamela Steffan ^4^, Jose L. Prado ^4^

*Yolanda Fernández-Jalvo (ORCID: 0000-0002-1089-7136)

**Email:** [yfj@mncn.csic.es](mailto:yfj@mncn.csic.es)

**This PDF file includes:**

Supporting text

Figures S1 to S4

Tables S1 to S10

SI References 59

Supporting Information Text

***The singular scenario of the Pampean Region.***

The Argentine Pampas, or Pampean Region, extend approximately between 30–39° S and 57–66° W, covering ~370,000 km². This vast plain, interrupted by the low hills of the Ventania and Tandilia ranges, forms a distinct biogeographic province characterized by a unique fauna and flora. The region accumulated Pleistocene sediments joining in few kilometers the presence of the paleontological site SPPL with megafauna killed by carnivores (no humans involved), close to other contemporaneous human occupation sites, with slaughtered megafaunal remains. This unusual situation allows us to obtain comparative data of the trophic pressure exerted by humans on the megafauna biodiversity, and human-carnivore interrelationship. The Pampean Region is subdivided today into the Humid Pampas to the southeast and the Dry Pampas to the west [1, 2]. Climatic fluctuations reflect the dynamic interplay between Pacific and Atlantic anticyclonic air masses: the Pacific Anticyclone, depleted of moisture after crossing the Andes, arrives cold and dry, while the Atlantic Anticyclone delivers warm, humid winds [3–5].

*Campo Laborde*: Evidence of Late Pleistocene Megafaunal Exploitation in the Pampas. This site (Fig. 1), located on a small tributary of the Tapalqué Creek, ~7 km south of SPPL (37º00’36” S, 60º23’05” W). Researchers interpret this site as a kill and butchering site for the giant ground sloth *Megatherium americanum*, dated to ~12,600 cal yr BP [6]. Additionally, lithic artifacts that excavators recovered near bones of glyptodonts (*Neosclerocalyptus*, *Doedicurus*), and extant taxa such as artiodactyls (Camelidae), Patagonian hare (*Dolichotis patagonum*), vizcacha (*Lagostomus maximus*), dwarf armadillo (*Zaedyus pichiy*), peccary (*Tayassu* sp.), fox (*Lycalopex* sp.), birds (Rheidae), and diverse microfauna from the same cultural deposit. Despite this diversity, unequivocal anthropogenic modifications have been documented exclusively on *Megatherium* bones [6].

*Arroyo Seco 2*: A Multi-Occupational Record of Human–Megafauna Interaction in the Pampas (Fig. 1), preserves the earliest human evidence in the region, dated to ~14,200 cal yr BP [7] and located about 180 km from SPPL (38°21’38” S, 60°14’39” W). Initial occupation phases document short-term carcass processing camps targeting *Equus neogeus* and *Megatherium americanum*. Another similar event occurred afterwards, where humans butchered two extinct horses (*Equus* sp. and *Hippidion* sp.) around 13,068 cal yrs BP. The site also yielded remains of other megamammals, such as *Toxodon platensis*, *Glossotherium robustum*, *Mylodon* sp., *Glyptodon* sp., *Macrauchenia* sp., Camelidae cf. *Hemiauchenia*, and Lestodontinae cf. *Lestodon* However, evidence of human agency on these bones remains ambiguous.

*Paso Otero 5*: Insights into Early Human Resilience and Mobility. Paso Otero 5 (Fig. 1) at 200 km from SPPL (38°12’08” S, 59°06’58” W) is the only Pampas site with a secure stratigraphic association between fishtail projectile points and megafaunal remains, dated between ~12,300 and 10,900 cal yr BP [8]. The faunal assemblage is highly fragmented and thermally altered, interpreted as raw material for fuel. Researchers identified at least 12 genera, including 10 extinct species. Among these, only *Hemiauchenia* sp. (<360 kg) exhibits potential anthropic fractures [9].

SPPL, near the city of Olavarría (Fig. 1; 36°56′54.6′′ S, 60°22′19.9′′ W), records past ecological environments without the involvement of contemporaneous human. SPPL corresponds to fluvial floodplain and lacustrine-palustrine environments [10] . The fossil association recorded in this site do not show any evidence of human artefacts or marks related to their activity. Among carnivores recorded in SPPL, *Smilodon populator* with an estimated body mass ranging from 220 to 400 kg [11, 12], possessed a hypercarnivorous diet and was highly specialized in hunting megaherbivores [13].

***Luminescence dating***

*Luminescence signals measured and sample preparation.*

Purified coarse-grained quartz was extracted from the luminescence samples under safe light (dim red LED) conditions at the University of Adelaide and prepared for burial dose estimation following established procedures [14]. For most samples, depositional ages were obtained by measuring the optically stimulated luminescence (OSL) signals from quartz grains [15]. However, K-feldspar fractions were extracted from lowermost samples SDP23-1 and SDP23-2 to measure their multi-grain post infrared (IR) IR stimulated luminescence (pIR-IRSL) signals [16], in order to cross-check the validity of the OSL ages, since these samples were potentially close to, or beyond, the upper age limit (saturation dose) of the OSL signal. Quartz thermally transferred (TT) OSL measurements [17] were also performed on these two lowermost samples as an additional cross-check of OSL dating suitability.

Sediment samples were initially sieved to isolate the fine sand fraction (90-300 µm) and treated with H_2_O_2_ and HCl to eliminate organics and carbonates, respectively. Heavy liquid separation (LST lithium heteropolytungstate) was used to isolate K-feldspar (2.53 to 2.58 g/cm^3^ density range) and quartz (2.62 to 2.72 g/cm^3^ density range) grains. The isolated K-feldspar 90-125 µm fraction was sieved and etched with 10% hydrofluoric acid for 10 minutes to remove the outer 10 µm rind of each grain [18]. Various quartz grain fractions were sieved out for single-grain OSL and TT-OSL burial dose estimation (see Table S1 for details of sample and grain size used) and subsequently etched with 48% hydrofluoric acid for 40 minutes to remove the alpha-irradiated external layers [16]. The etched quartz and K-feldspar grains were then washed in 30% hydrochloric acid to remove any precipitated fluorides and re-sieved using a 63 µm sieve to eliminate any disaggregated grains.

***Methodological remarks***

Dose rates: Environmental dose rates have been determined using high-resolution gamma-ray spectroscopy (HRGS) (Table S1). HRGS measurements were made on dried and powdered sediment samples collected immediately adjacent to the luminescence dating samples using a p-type, high-purity, germanium well detector, following the procedures outlined in Leslie [19]. For each sample, ~7 g of dried and powdered bulk sediment was sealed in a plastic container for at least 30 days (the equivalent of ~8 half-lives of ^222^Rn; t_1/2_ = 3.825 days) to enable the post-radon daughters of ^214^Pb and ^214^Bi to build up and reach equilibrium with parental ^226^Ra activities. Following re-establishment of equilibrium in the post-radon nuclides, the sealed samples were counted in the well detector for 3–4 days, in near-4π geometry to maximise detection efficiency. The specific activities of ^238^U (determined from ^235^U emissions after correcting for ^226^Ra interference, and ^234Th^ emissions after correcting for ^228^Ra interference), ^226^Ra (derived from ^214^Pb and ^214^Bi emissions), ^210^Pb, ^228^Ra (derived from ^228^Ac emissions), ^228Th^ (derived from ^212^Pb, ^212^Bi and ^208^Tl emissions) and ^40^K were measured for each sediment sample, and used to derive daughter-to-parent isotope ratios for ^226^Ra:^238^U, ^210^Pb:^226^Ra and ^228Th^:^228^Ra.

External beta and gamma dose rates were then calculated from the HRGS radionuclide activities using the conversion factors of Guèrin [20], making allowance for beta-dose attenuation [21, 22] and long-term sediment water contents [23, 24]. The present-day sediment water contents, which ranged between ~5% and ~24% (though most values were 18–24%), are considered representative of long-term moisture conditions (Table S1). A 1σ relative uncertainty of ±20% (±40% at 2σ) was assigned to these long-term estimates to account for any past variations in hydrological conditions during burial. Representative dose rates have been calculated from the HRGS radionuclide activities after considering the proportional beta and gamma dose rate contributions from different parent or daughter isotopes measured in the ^238^U and ^232^Th decay series. As part of this analysis, the present-day state of (dis)equilibrium observed in the ^238^U and ^232^Th decay chains is assumed to have prevailed throughout the burial period.

Cosmic ray contributions have been calculated following Prescott and Hutton [25]. An internal ^40^K content of 12.5 ± 0.5% [26] and ^87^Rb content of 400 ± 100 ppm [27] has been assumed for determining the internal dose rates of K-feldspar grains. An internal dose date of 0.03 Gy/ka was assumed for quartz grains following previous publications (see footnote ‘e’ in Table S1).

The HRGS radionuclide activities obtained for the seven luminescence dating samples are summarized in Table S2. The daughter-parent isotopic ratios for ^238^U, ^226^Ra, ^210^Pb, ^228^Ra and ^228Th^ are consistent with unity at either 1σ or 2σ in most instances, confirming that the ^238^U and ^232^Th chains exhibit present-day secular equilibrium (Table S2). However, the upper three samples from U3 and U4 (SDP23-6, SDP23-4 and SDP23-5) produced ^226^Ra:^238^U ratios of 0.5-0.6 and indicate moderate disequilibrium in the initial part of the ^238^U chain, which may suggest recent incorporation of soluble ^238^U into these sediment layers. The cause of this uranium series disequilibrium was investigated by assessing the lithogenic ^230Th^:^232^Th ratios for these three samples (approximated from the measured ^238^U:^228^Ra ratios, assuming equilibrium between ^238^U and ^230Th^ at the head of the uranium decay series, and assuming equilibrium between ^232^Th and ^228^Ra at the head of the thorium decay series). The deposits at Salto de Piedra may be expected to have broadly similar ^230Th^:^232^Th ratios, all else being equal, if they originated from a common sediment source (see justification in Olley [28]. Samples SDP23-7, SDP23-3, SDP23-2 and SDP23-1 exhibit a relatively narrow range of ^230Th^:^232^Th (^238^U:^228^Ra) ratios (0.7 to 1.2). In contrast, samples SDP23-5, SDP23-4 and SDP23-6 exhibit significantly higher ^238^U:^228^Ra values (1.5-2.5), consistent with the uptake of additional, unsupported parental ^238^U at the head of the uranium decay series. This interpretation is also supported by considering the ^226^Ra:^228^Ra ratios of samples SDP23-5, SDP23-4 and SDP23-6 as a proxy for their lithogenic ^230Th^:^232^Th ratios. Using this alternative assessment, the three samples yield ^230Th^:^232^Th ratios of 0.9-1.3, which are in closer agreement with the ^230Th^:^232^Th ratios of the samples that are in present-day secular equilibrium. Irrespective, the excess ^238^U activities of samples SDP23-5, SDP23-4 and SDP23-6 are not considered to have a sig^ni^fica^nt^ effect on the final dose rate estimates, as inferred from the stratigraphic consistency of the single-grain OSL ages obtained for the sediment sequence at Salto de Piedra and their overall agreement with independent age control (^14^C chronologies). Dosimetry modelling studies undertaken elsewhere have demonstrated that isotopic disequilibria of similar magnitudes are only likely to give rise to minor deviations (<5%) in long-term dose rate estimates [29–31]. Such systematic biases would be significantly less than the existing uncertainty ranges on our final dose rate estimates. The effects of ^238^U disequilibrium will be further diminished for these samples because the ^238^U decay series contributes <30% to the total quartz dose rate (calculated using the high-resolution gamma spectrometry results).

Luminescence measurement instrumentation: Several Risø TL/OSL-DA-20 readers with dual laser single-grain attachments, EMI 9235QB or Electron Tubes PDM 9107B photomultiplier tubes, and spatially calibrated ^90^Sr / ^90^Y β sources (~3–8.6 Gy / min) were used for measuring single-grain OSL, single-grain TT-OSL and multi-grain pIR-IRSL signals. OSL and TT-OSL measurements were made on quartz grains of various sizes, either 90-125 μm, 125-250 μm or 212-250 μm, which were loaded into single-grain aluminum discs with 300 µm-deep holes. The OSL signals were stimulated using a green laser (532 nm) and UV emissions were detected through a 7.5 mm-thick U-340 Hoya filter. pIR-IRSL signals were measured using K-feldspar-rich grain fractions that had been mounted as monolayers on stainless steel discs (90-125 μm fractions, ~360 grains on each disc). pIR-IRSL signals were stimulated using an IR diode array (peak emission 870 nm), with blue emissions measured through a BG39 (4 mm), 7-59 (3 mm) and GG400 (3 mm) filter pack.

Equivalent dose (D_e_) determination: Single-grain OSL, single-grain TT-OSL and multi-grain pIR-IRSL D_e_ values were obtained using the single-aliquot regenerative-dose (SAR) protocols shown in Table S3. These protocols represent modified versions of those published in recent years [15-17]. OSL net L_x_ and T_x_ values were calculated by integrating the first 0.09 s of the decay curves and subtracting a background count from the last 0.25 s of the signal. TT-OSL net L_x_ and T_x_ values were calculated by integrating the first 0.18 s of the decay curves and subtracting a background count from the last 0.25 s of the signal. Multi-grain pIR-IRSL D_e_ values were calculated from the first 10 s of stimulation after subtracting a mean background count from the last 20 s of stimulation. The OSL and TT-OSL SAR dose-response curves were fitted using a single saturating exponential function, whilst the pIR-IRSL dose-responses were fitted using a saturating exponential plus linear function (examples of these are shown in Fig. S1).

Individual D_e_ values were accepted if they passed the following quality assurance criteria: (i) the luminescence signals were statistically distinguishable from background (the net intensity of the natural test dose signal, T_n_, was >3σ above the late-light background signal); (ii) the recycling ratio (i.e., sensitivity-corrected luminescence responses (L_x_/T_x_) for two identical high regenerative doses) was consistent with unity at 2σ; (iii) the recuperation ratio, calculated as the ratio of the sensitivity-corrected 0 Gy dose point (L_0_/T_x_) to the sensitivity-corrected natural (L_n_/T_n_), was <5%; (iv) the OSL IR depletion ratio of Duller [31] was consistent with unity at 2σ (i.e., the ratio of the L_x_/T_x_ values obtained for two identical regenerative doses measured with and without prior IR stimulation) – this criterion is designed to detect feldspar contamination or inclusions and was only applied to the OSL and TT-OSL measurements; (v) the net T_n_ signal had a relative error of <30%; (vi) the sensitivity-corrected natural signal intercepted the sensitivity-corrected dose-response curve and the intercept point was below the saturated part of the dose-response curve (i.e., the L_n_/T_n_ value was lower than the *I_max_* saturation limit of the dose-response curve at 2σ); (vii) the dose-response curve did not display anomalous properties (e.g., non-monotonic, zero (flat) or negative responses with increasing dose) and resulted in suitable Monte Carlo fits. The OSL and TT-OSL grain classification statistics obtained for each sample after applying these quality assurance criteria are summarized in Table S4. Multi-grain aliquot pIR-IRSL measurements were subject to the same quality assurance criteria as the OSL measurements, with the exception of criterion (iv). All of the measured K-feldspar aliquots passed these quality assurance criteria and were included in the final D_e_ analysis.

Individual D_e_ estimates are presented with their 1σ error ranges, which are derived from three sources of uncertainty: (i) a random uncertainty term arising from photon counting statistics for each OSL or pIR-IRSL measurement, calculated using Eq. 3 [32, 33] (ii) an empirically determined instrument reproducibility uncertainty of 0.6% for each multi-grain aliquot measurement and 1.9% for each single-grain measurement (calculated for the specific Risø reader used for each sample[34]; and (iii) a dose-response curve fitting uncertainty determined using 1000 iterations of the Monte Carlo method [15, 35] and implemented in Analyst v4.

Single-grain OSL dose recovery tests: Single-grain OSL dose recovery test measurements were made on quartz grains from sample SDP23-6 by administering a known dose of 55 Gy to grains that had previously been bleached with two exposures of 1,000 s to blue LEDs, separated by a 10,000 s pause. Regenerative dose and test dose preheat treatments of 240°C for 10 s and 200°C for 10 s, respectively, were used in the single-grain dose recovery test (protocol A; Table S3). These measurement conditions were chosen because these were previously considered optimal for the Salto de Piedra samples [36]. A single-grain OSL measured-to-given dose ratio of 1.00 ± 0.04 and a corresponding overdispersion value of 2 ± 1 % was obtained using this procedure. This dose recovery ratio is within 2σ of unity and supports the suitability of the D_e_ measurement conditions used in this study.

Single-grain TT-OSL dose recovery tests: The single-grain TT-OSL dose recovery test was performed on sample SDP23-1 after administering a dose of 396 ± 2 Gy on top of the naturally accumulated dose for a sub-set of grains and measuring the resulting doses using SAR protocol C in Table S3 [37]. The dose recovery ratio was calculated by first adding the weighted mean natural TT-OSL D_e_ of sample SDP23-1 (594 ± 36 Gy) to the weighted mean administered dose of the accepted grain population from the dose recovery test (396 ± 2 Gy). This was used to constrain the combined administered (natural + laboratory) dose being assessed in the dose recovery test. The measured (recovered) weighted mean D_e_ of the unbleached and dosed grains in the dose recovery test (1095 ± 95 Gy) was then divided by the combined administered (natural + laboratory) dose (991 ± 36 Gy) to derive the measured-to-given dose ratio (see Table S4 for details). A dose recovery ratio of 1.10 ± 0.10 was obtained using this approach, which is within 2σ of unity and supports the suitability of the chosen measurement conditions for dating these samples.

Multi-grain pIR-IRSL dose recovery tests: pIR-IRSL dose recovery tests were undertaken on sample SDP23-2 after bleaching the naturally accumulated signals of two batches of five K-feldspar aliquots (each containing 360-grains) in direct sunlight for 8 hrs. For each batch of 5 aliquots, two were left un-dosed to determine the residual (unbleached) D_e_ remaining after daylight exposure, while the remaining three aliquots were given a laboratory dose of 650 ± 13 Gy (Table S5). The first batch of five aliquots was then measured with pIR-IRSL_225_ protocol C shown in Table S3 to determine their D_e_ values. The dose recovery (measured to given dose) ratio was calculated after subtracting the residual (unbleached) D_e_ of the un-dosed aliquots from the weighted mean D_e_ obtained from the dosed aliquots (Table S5). The pIR-IRSL_225_ dose recovery ratio overlaps with unity at 2σ (ratio is 0.97 ± 0.03; Table S5) and support the suitability of the SAR protocol for D_e_ determination. The same test was performed on the second batch of five aliquots but using a preheat of 320°C for 60 seconds and measuring the pIR-IRSL signal at 290°C (pIR-IRSL_290_). These alternative measurement conditions did not produce an accurate dose recovery ratio and are therefore not considered suitable for these samples (dose recovery ratio is 1.13 ± 0.03; Table S5). Accordingly, samples SDP23-1 and SDP23-2 from Salto de Piedra were dated using pIR-IRSL_225_ signals.

pIR-IRSL_225_ anomalous fading tests: Assessments for anomalous fading rates were performed on subsets of aliquots for samples SDP23-2 and SDP23-1 to investigate the potential for long-term athermal loss of the K-feldspar pIR-IRSL_225_ signal over burial timescales. To achieve this, we applied the procedure of Auclair et al. [38], which involves undertaking repeated SAR L_x_/T_x_ measurements after delayed storage times of 0.2 to 30 h. Anomalous fading rates (g-values) normalized to 2 days were calculated [27] and used to quantify the expected percentage of signal loss per decade of storage time (Table S6).

The results of these anomalous fading tests are presented in Table S7 and indicate fading rates values of ~1.90 %/decade for sample SDP23-2 and ~3.5 %/decade for sample SDP23-1. The average g-value for these samples (2.56 ± 0.46) is higher than the 1–2 %/decade range commonly reported for pIR-IRSL_225_ and pIR-IRSL_290_ datasets that are considered athermally stable [19, 39]. Therefore, we have applied an empirical fading correction to the pIR-IRSL_225_ ages obtained for samples SDP23-1 and SDP23-2 using the measured g-values and the procedure of Huntley and Lamothe [27].

***Luminescence dating - Results***

D_e_ distribution: The calculated D_e_ values, overdispersion values (i.e., the degree of scatter beyond the empirical D_e_ uncertainties) and ages for the OSL, TT-OSL, and pIR-IRSL_225_ signals of the Salto de Piedra samples are summarized in Tables S8, S9, and S10, respectively. For each sample and signal type, the D_e_ distribution has been analysed using the 3-parameter and 4-parameter minimum age models (MAM-3 and MAM-4, respectively) and the central age model (CAM) described in Galbraith et al. [40]. The MAM-3, MAM-4, and the weighted skewness test were applied after adding, in quadrature, a relative error of 20% to each individual D_e_ measurement error to approximate the underlying dose overdispersion observed in ‘ideal’ (well-bleached and unmixed) sedimentary samples elsewhere [42]. The suitability of age model fitting was assessed using the maximum log likelihood ratio criterion (*L_max_* score) [41] (see footnote ‘d’ in Table S8 for details on interpretation of *L_max_* values). The corresponding De distributions for each sample are shown as radial plots in Figs. S1-S3.

Single-grain OSL measurements were made on all samples except for the lowermost sample SDP23-1. All D_e_ distributions present low to moderate overdispersion, ranging from ~24% to ~33%, and none of the samples display the signature effects of partial bleaching; i.e., they are not considered to be significantly positively skewed according to the weighted skewness test and their *L_max_* scores support the suitability of the CAM fitting over the MAM-3 and MAM-4 fitting (see Table S8; footnotes ‘a’ and ‘c’). Therefore, the weighted mean OSL De values for all samples were calculated using the CAM (Table S8 and Fig. S1).

The CAM single-grain OSL D_e_ of sample SDP23-2, from the lowermost Unit 1 Facie 3 (U1-F3), is 257.0 ± 18.5 Gy, which is significantly higher than for the rest of the samples, and closer to the saturation limit of the OSL signal. To examine whether this OSL dataset may be affected by dose saturation issues, and therefore represents a minimum rather than a finite age estimate for sample SDP23-2, we undertook replicate luminescence dating using the K-feldspar pIR-IRSL_225_ signal and the single-grain quartz TT-OSL signal, both of which have higher dose-saturation limits than conventional OSL. These two extended-range luminescence signals were also measured for sample SDP23-1, which was similarly collected from U1-F3. The pIR-IRSL_225_ and TT-OSL D_e_ distributions for these two samples are relatively homogeneous, exhibit low to moderate overdispersion, and statistically favor the use of the CAM over the MAM-3 or MAM-4 (Fig. S2 and Fig. S3). For both SDP23-1 and SDP23-2, which have similar dose rate characteristics, the CAM TT-OSL D_e_ values are ~590 Gy (Table S9 and Fig. S2) and the pIR-IRSL_225_ D_e_ values are ~360-570 Gy (Table S10 and Fig. S4), respectively. These extended-range luminescence D_e_ values are significantly higher than the corresponding weighted mean D_e_ values obtained using single-grain OSL for SDP23-2 (~257 Gy) (Table S9 and Fig. S1) and confirm that the naturally accumulated dose of sample SDP23-2 is too high to be reliably measurable using conventional OSL D_e_ estimation procedures. As such, for these lowermost samples, the TT-OSL and pIR-IRSL_225_ burial doses are considered to provide the most accurate age estimates, with the single-grain OSL age of SDP23-2 interpreted as a minimum age for U1-F3.

Final ages: The single-grain OSL ages obtained for the Salto de Piedra sequence are shown in Table S8. The additional TT-OSL and pIR-IRSL_225_ ages obtained for U1 are shown in Table S9 and Table S10, respectively. In these tables, the preferred ages are shown in bold and marked with an asterisk. The ages obtained are in stratigraphic order and range from ~60 ka to ~14 ka for U2 to U4, whilst the lower sample from U1 produced a single-grain OSL minimum age estimate of >124 ka. The application of extended-range luminescence dating (TT-OSL and pIR-IRSL_225_) on two samples from U1 produced ages ranging from ~295 ka to ~185 ka. The replicate TT-OSL and pIR-IRSL_225_ ages of each sample overlap at 2σ and are therefore considered statistically indistinguishable. Given that the naturally accumulated single-grain OSL signal of SDP23-2 is in saturation (OSL D_e_ ≈ 260 Gy), it is considered that U1 formed >124 ka, and most likely around 245.6 ± 23.7 ka (corresponding to the combined weighted mean TT-OSL and pIR-IRSL_225_ age obtained for samples SDP23-1 and SDP23-2).

**Fig. S1.** Examples of (A) OSL, (B) TT-OSL, and (C) pIR-IRSL_225_ decay curves and sensitivity‐corrected dose–response curves (inset) for representative quartz grains of samples SDP23-3 and SDP23-1 and a multi-grain aliquot of K-feldspars from sample SDP23-2, respectively. White square denotes the sensitivity-corrected natural signal; filled circles denote the sensitivity‐corrected regenerative dose signals; white circles denote the repeated regenerative dose points used to calculate the recycling ratios. The D_0_ value characterizes the rate of signal saturation with respect to administered dose and equates to the dose value for which the saturating exponential dose–response curve slope is 1/e (or ~0.37) of its initial value.

**Fig. S2**. Single-grain OSL D_e_ distributions for the Salto de Piedra samples shown as radial plots. Grey bars are centred on the CAM D_e_ values.

**Fig. S3.** Single-grain TT-OSL D_e_ distributions for the Salto de Piedra samples SDP23-1 and SDP23-2 shown as radial plots. Grey bars are centred on the CAM D_e_ values.

**Fig. S4.** Multi-grain pIR-IRSL_225_ D_e_ distributions for the Salto de Piedra samples SDP23-1 and SDP23-2 shown as radial plots. Grey bars are centred on the CAM D_e_ values.

| **Sample** | **Unit** | **Facie** | **Sample depth**  **(cm)** | **Water content**  **(%) ^a^** | **Mineral** | **Dating method** | **Grain**  **size**  **(μm)** | **Environmental dose rate (Gy/ka)** | | | | |
| --- | --- | --- | --- | --- | --- | --- | --- | --- | --- | --- | --- | --- |
|  |  |  |  |  |  |  |  | **Gamma ^b^** | **Beta ^b,c^** | **Cosmic ^d^** | **Internal ^e^** | **Total ^f^** |
| SDP23-5 | 4 | 9 | 160 | 19.5 | quartz | OSL | 180-250 | 0.93 ± 0.01 | 1.41 ± 0.07 | 0.15 ± 0.02 | 0.03 ± 0.01 | 2.53 ± 0.10 |
| SDP23-4 | 4 | 8 | 280 | 4.8 | quartz | OSL | 180-250 | 1.31 ± 0.02 | 1.97 ± 0.10 | 0.15 ± 0.02 | 0.03 ± 0.01 | 3.48 ± 0.12 |
| SDP23-6 | 3 | 6 | 360 | 22.1 | quartz | OSL | 90-125 | 1.03 ± 0.01 | 1.55 ± 0.08 | 0.12 ± 0.01 | 0.03 ± 0.01 | 2.72 ± 0.11 |
| SDP23-7 | 3 | 5 | 480 | 18.9 | quartz | OSL | 90-125 | 0.96 ± 0.01 | 1.61 ± 0.08 | 0.11 ± 0.01 | 0.03 ± 0.01 | 2.71 ± 0.11 |
| SDP23-3 | 2 | 4 | 620 | 16.7 | quartz | OSL | 212-250 | 0.81 ± 0.01 | 1.19 ± 0.06 | 0.09 ± 0.01 | 0.03 ± 0.01 | 2.12 ± 0.08 |
| SDP23-2 | 1 | 3 | 670 | 18.8 | quartz | OSL | 90-125 | 0.78 ± 0.01 | 1.18 ± 0.06 | 0.09 ± 0.01 | 0.03 ± 0.01 | 2.08 ± 0.08 |
|  |  |  |  |  | quartz | TT-OSL | 125-250 | 0.78 ± 0.01 | 1.12 ± 0.06 | 0.09 ± 0.01 | 0.03 ± 0.01 | 2.02 ± 0.08 |
|  |  |  |  |  | K-feldspar | pIR-IRSL_225_ | 90-125 | 0.78 ± 0.01 | 1.18 ± 0.06 | 0.09 ± 0.01 | 0.49 ± 0.03 | 2.54 ± 0.09 |
| SDP23-1 | 1 | 3 | 700 | 24.0 | quartz | TT-OSL | 90-125 | 0.87 ± 0.01 | 1.33 ± 0.07 | 0.08 ± 0.01 | 0.03 ± 0.01 | 2.31 ± 0.10 |
|  |  |  |  |  | K-feldspar | pIR-IRSL_225_ | 90-125 | 0.87 ± 0.01 | 1.33 ± 0.07 | 0.08 ± 0.01 | 0.49 ± 0.03 | 2.77 ± 0.10 |

^a^ Long-term water content is expressed as % of dry mass of mineral fraction and assigned relative uncertainties of ±20%.

^b^ Gamma and beta dose rates were calculated from sample-specific radionuclid[42] activities of U, Th and K determined from HRGS measurements (Table S2).

^c^ Beta dose rates have been calculated after making allowance for beta dose attenuation due to grain-size effects and HF etching [43].

^d^ Cosmic-ray dose rates were calculated using the approach of Prescott and Hutton [25] and assigned a relative uncertainty of ±10%.

^e^ An internal dose rate of 0.03 Gy/ka, with an assigned relative uncertainty of ±30%, has been adopted for the quartz fractions of these samples based on intrinsic ^238^U and ^232^Th contents published by Mejdahl [21], Bowler [44], Jacobs [45], Pawley [46] and Lewis [47], and an a-value of 0.04 ± 0.01 [48, 49]. Internal dose rates for feldspar grains arising from ^40^K and ^87^Rb concentrations were calculated using assumed values of 12.5 ± 0.5% (*62*) and 400 ± 100 ppm [42], respectively. Internal ^238^U and ^232^Th concentrations were assumed to be 0.15 ± 0.03 ppm and 0.35 ± 0.07 ppm, respectively, based on modal values obtained by Mejdahl [50] and similar values obtained by Huntley and Clague [51], Huntley and Lian [52], and Alappat [53]. An a-value of 0.09 ± 0.03 was used to estimate the internal alpha dose rate contributions from these ^238^U and ^232^Th concentrations based on published estimates obtained for a wide range of K-feldspar samples [54].

^f^ Mean ± total uncertainty (68% confidence interval), calculated as the quadratic sum of the random and systematic uncertainties.

**Table S1.** Environmental dose rate summary for the single-grain quartz OSL, single-grain TT-OSL and multi-grain pIR-IRSL_225_ samples from Salto de Piedra, Olavarría city, Buenos Aires province, Argentina.

| **Sample** |  | **Radionuclide specific activities (Bq/kg) ^a, b^** | | | | | |  | **Daughter: parent isotopic ratio** | | |
| --- | --- | --- | --- | --- | --- | --- | --- | --- | --- | --- | --- |
|  |  | **^238^U** | **^226^Ra** | **^210^Pb** | **^228^Ra** | **^228^Th** | **^40^K** |  | **^226^Ra:^238^U** | **^210^Pb:^226^Ra** | **^228^Th:^228^Ra** |
| SDP23-5 |  | 50.8 ± 3.1 | 32.4 ± 0.7 | 32.9 ± 3.0 | 33.6 ± 1.3 | 36.2 ± 1.0 | 554 ± 14 |  | 0.64 ± 0.04 | 1.02 ± 0.10 | 1.08 ± 0.05 |
| SDP23-4 |  | 100.4 ± 3.3 | 53.6 ± 0.8 | 54.8 ± 3.5 | 40.6 ± 1.2 | 40.4 ± 1.0 | 563 ± 14 |  | 0.53 ± 0.02 | 1.02 ± 0.07 | 0.99 ± 0.04 |
| SDP23-6 |  | 62.2 ± 2.7 | 40.6 ± 0.7 | 43.6 ± 2.8 | 42.3 ± 2.8 | 43.7 ± 1.0 | 542 ± 13 |  | 0.65 ± 0.03 | 1.07 ± 0.07 | 1.04 ± 0.04 |
| SDP23-7 |  | 39.8 ± 2.4 | 33.2 ± 0.6 | 26.6 ± 2.8 | 36.9 ± 1.2 | 38.4 ± 1.0 | 554 ± 14 |  | 0.83 ± 0.05 | 0.80 ± 0.09 | 1.04 ± 0.04 |
| SDP23-3 |  | 33.3 ± 2.4 | 32.3 ± 0.6 | 30.7 ± 3.0 | 27.7 ± 1.1 | 29.3 ± 0.8 | 439 ± 12 |  | 0.97 ± 0.07 | 0.95 ± 0.10 | 1.06 ± 0.05 |
| SDP23-2 |  | 25.7 ± 2.4 | 27.9 ± 0.6 | 22.2 ± 2.7 | 32.0 ± 1.2 | 31.5 ± 0.9 | 433 ± 12 |  | 1.09 ± 0.11 | 0.79 ± 0.10 | 1.02± 0.05 |
| SDP23-1 |  | 29.5 ± 2.5 | 26.8 ± 0.5 | 28.6 ± 2.8 | 41.4 ± 1.1 | 42.1 ± 1.0 | 491 ± 12 |  | 0.91 ± 0.08 | 1.07 ± 0.11 | 1.02 ± 0.04 |

^a^ Measurements made on dried and powdered sediment subsamples of ~7 g.

^b^ Mean ± total uncertainty (68% confidence interval), calculated as the quadratic sum of the random and systematic uncertainties.

**Table S2.** High-resolution gamma-ray spectrometry (HRGS) results for luminescence dating samples from Salto de Piedra, Olavarría city, Buenos Aires province, Argentina.

| **A** | **Single-grain OSL** |  |  | **B** | **Single-grain TT-OSL** |  |  | **C** | **Single aliquot pIR-IRSL_225_** |  |
| --- | --- | --- | --- | --- | --- | --- | --- | --- | --- | --- |
| **Step** | **Treatment** | **Signal** |  | **Step** | **Treatment** | **Signal** |  | **Step** | **Treatment** | **Signal** |
| 1^a^ | Give dose |  |  | 1^a^ | Give dose |  |  | 1^a^ | Give dose |  |
| 2 | Preheat to 240ºC for 10 s |  |  | 2 | Preheat to 260ºC for 10 s |  |  | 2 | Preheat to 250ºC for 60 s |  |
| 3 | Stimulate with green laser at 125ºC for 2 s | OSL  L_n_ or L_x_ |  | 3 | Stimulate with green laser at 125ºC for 2 s |  |  | 3 | Stimulate with IR LEDs at 50ºC for 200 s |  |
| 4 | Give test dose (15 Gy) |  |  | 4 | Preheat to 260ºC for 10 s |  |  | 4 | pIR-IRSL stimulation at 225ºC for 200 s | pIR-IRSL_225_  L_n_ or L_x_ |
| 5 | Preheat to 200ºC for 10 s |  |  | 5 | Stimulate with green laser at 125ºC for 3 s | TT-OSL  L_n_ or L_x_ |  | 5 | Give test dose (100 Gy) |  |
| 6 | Stimulate with green laser at 125ºC for 2 s | OSL  T_n_ or T_x_ |  | 6 | Stimulate with blue LEDs at 280ºC for 400 s |  |  | 6 | Preheat to 250ºC for 60 s |  |
| 7 | Return to 1 |  |  | 7 | Give test dose (200 Gy) |  |  | 7 | Stimulate with IR LEDs at 50ºC for 200 s | pIR-IRSL_225_  T_n_ or T_x_ |
|  |  |  |  | 8 | Preheat to 260ºC for 10 s |  |  | 8 | pIR-IRSL stimulation at 225ºC for 200 s |  |
|  |  |  |  | 9 | Stimulate with green laser at 125ºC for 2 s |  |  | 9 | High temperature IR wash at 290ºC for 100 s |  |
|  |  |  |  | 10 | Preheat to 260ºC for 10 s |  |  | 10 | Return to 1 |  |
|  |  |  |  | 11 | Stimulate with green laser at 125ºC for 3 s | TT-OSL  T_n_ or T_x_ |  |  |  |  |
|  |  |  |  | 12 | Stimulate with blue LEDs at 290ºC for 400 s |  |  |  |  |  |
|  |  |  |  | 13 | Return to 1 |  |  |  |  |  |

^a^ Step omitted when measuring the natural signal (L_n_).

**Table S3.** SAR protocols used in this study to obtain single-grain OSL (protocol A), single-grain TT-OSL (protocol B) and pIR-IRSL_225_ (protocol C) D_e_ estimates. L_n_ and L_x_ refer to the natural and regenerative-dose signal measurements, respectively. T_n_ and T_x_ refer to the test dose signals measured after the L_n_ and L_x_ signals, respectively.

| **Sample** |  | **Grains measured (n)** |  |  |  |  | **Rejected (%)** | |  |  |  | **Accepted grains (%)** |
| --- | --- | --- | --- | --- | --- | --- | --- | --- | --- | --- | --- | --- |
|  |  |  | **T_n_ signal <3xBG** | **Poor recycling ratio (low)** | **Poor recycling ratio (high)** | **IR depletion ratio** | **Recuperation >5%** | **Net T_n_ error >30%** | **L_n_/T_n_ not intercepting DRC** | **Anomalous dose-response curve** | **Saturated** |  |
| D_e_ estimation |  |  |  |  |  |  |  |  |  |  |  |  |
| SDP23-5 | OSL | 2000 | 76.3 | 7.6 | 2.8 | 2.1 | 0.2 | 6.5 | 0.1 | 0.7 | 0.1 | **3.7** |
| SDP23-4 | OSL | 200 | 82.0 | 6.0 | 1.5 | 1.0 | 0 | 3.0 | 1.0 | 0 | 0.5 | **5.0** |
| SDP23-6 | OSL | 5100 | 79.5 | 10.1 | 3.3 | 1.1 | 0.1 | 3.1 | 0.1 | 0.3 | 0.3 | **2.1** |
| SDP23-7 | OSL | 1900 | 63.1 | 7.3 | 3.7 | 6.9 | 0.2 | 8.1 | 0.5 | 0.8 | 1.3 | **8.1** |
| SDP23-3 | OSL | 2400 | 41.5 | 6.8 | 5.3 | 5.0 | 0.8 | 16.7 | 1.8 | 7.1 | 5.0 | **10.0** |
| SDP23-2 | OSL | 300 | 44.0 | 8.0 | 5.3 | 4.3 | 1.0 | 6.7 | 5.3 | 8.7 | 9.7 | **7.0** |
| SDP23-2 | TT-OSL | 800 | 76.0 | 8.3 | Not measured | 0 | 0 | 13.5 | 0 | 0 | 0.1 | **2.1** |
| SDP23-1 | TT-OSL | 2400 | 56.5 | 6.8 | Not measured | 6.8 | 0.1 | 25.3 | 0.3 | 1.1 | 0.1 | **3.8** |
| Dose recovery tests |  |  |  |  |  |  |  |  |  |  |  |  |
| SDP23-6 | OSL | 1400 | 80.4 | 9.2 | 2.8 | 1.1 | 0 | 3.3 | 0.1 | 0.1 | 0.1 | **2.2** |
| SDP23-1 | TT-OSL | 500 | 57.8 | 7.2 | Not measured | 4.0 | 0 | 24.8 | 0.8 | 1.4 | 0 | **4.0** |

**Table S4.** Single-grain OSL and TT-OSL classification statistics showing proportion of rejected and accepted grains after applying the SAR quality assurance criteria to the natural D_e_ measurements and dose recovery test measurements. These criteria were applied to each single-grain measurement in the order shown (from left to right). T_n_ = natural test dose signal response; L_n_/T_n_ = sensitivity-corrected natural signal response; DRC = dose response curve; BG = background signal.

|  | **Natural dose assessment (un-dosed grains)** | | **Dose recovery test results** | | | | **Measured / given dose ratio (natural dose +given dose)** |
| --- | --- | --- | --- | --- | --- | --- | --- |
| **Sample** | **Accepted / Measured** | **CAM D_e_ (Gy) ^a^** | **Given dose (Gy)** | **Accepted / measured** | **CAM D_e_ (Gy) ^a^** | **OD (%) ^a^** |  |
| SDP23-1 | 90/2400 | 594 ± 36 | 396 ± 2 | 20 / 500 | 1095 ± 95 | 23 ± 10 | 1.10 ± 0.10 |

^a^ Mean ± total uncertainty (68% confidence interval), calculated as the quadratic sum of the random and systematic uncertainties.

**Table S5.** Single-grain TT-OSL dose recovery test results for 90-125 µm quartz grains of sample SDP23-1.

| **Sample** | **Mineral/**  **Signal** | **Grains per disc** | **Grain size (µm)** | **Given dose (Gy)** | **Bleaching** | **Residual (non-dosed) assessment** | | | **Dose recovery test** | | | |
| --- | --- | --- | --- | --- | --- | --- | --- | --- | --- | --- | --- | --- |
|  |  |  |  |  |  | **accepted/measured** | **Weighted mean D_e_ (Gy)** | **Over-dispersion (%)** | **accepted/**  **measured** | **Weighted**  **mean D_e_**  **(Gy)** | **Over-dispersion (%)** | **Net measured/**  **given dose**  **ratio ^a^** |
| SDP23-2 | K-feldspar /  pIR-IRSL_225_ | ~360 | 90-125 | 650 ± 13 | 8 hours in daylight | 2 / 2 | 10 ± 1 | 9 ± 5 | 3 / 3 | 643 ± 18 | 4 ± 2 | 0.97 ± 0.03 |
| SDP23-2 | K-feldspar /  pIR-IRSL_290_ | ~360 | 90-125 | 650 ± 13 | 8 hours in daylight | 2 / 2 | 22 ± 4 | 25 ± 13 | 3 / 3 | 758 ± 16 | 2 ± 3 | 1.13 ± 0.03 |

^a^ Mean ± total uncertainty (68% confidence interval), calculated as the quadratic sum of the random and systematic uncertainties.

**Table S6.** Multi-grain aliquot dose recovery test results for the K-feldspar pIR-IRSL_225_ and pIR-IRSL_290_ signals of sample SDP23-2.

| **Sample** | **Fading rate (*g_2days_*)**  **(% / decade) ^a^** |
| --- | --- |
| SDP23-2 (*n* = 4 aliquots) | 1.90 ± 0.42 |
| SDP23-1 (*n* = 3 aliquots) | 3.43 ± 0.67 |
| AVERAGE (*n* = 7 aliquots) | 2.56 ± 0.46 |

^a^ Mean ± total uncertainty (68% confidence interval), calculated as the quadratic sum of the random and systematic uncertainties. Total uncertainty includes a systematic component of ± 2% associated with laboratory beta-source calibration.

**Table S7.** K-feldspar pIR-IRSL_225_ empirical fading rates (g-values) for the Salto de Piedra samples. The *g*-values were determined from repeated L_x_/T_x_ measurements made after different storage times (ranging from 0.2 h to 30 h) using Eq. 4 [27], and have been normalised to a measurement delay time of two days (*g_2days_*) to enable direct comparisons with published values.

| **Sample** | **Sample**  **depth**  **(cm)** | **Level** | **Grain**  **size**  **(μm)** | **Total**  **dose rate**  **(Gy/ka)** | **Accepted/Measured** | **Overdis-persion (%)** | **Weighted skewness ^a^** | **Critical skewness**  **(95% C.I.)  ^a^** | **Critical skewness**  **(68% C.I.)  ^a^** | **Age Model**  **^b, c^** | ***L_max_* score ^d^** | **D_e_ (Gy) ^e^** | **Age (ka) ^e, f^** |
| --- | --- | --- | --- | --- | --- | --- | --- | --- | --- | --- | --- | --- | --- |
| SDP23-5 | 160 | U4 – F9 | 180-250 | 2.53 ± 0.10 | 76 / 2000 | 24 ± 4 | -0.322 | 0.561 | 0.280 | CAM | -24.735 | 35.6 ± 1.4 | **14.1 ± 0.8*** |
|  |  |  |  |  |  |  |  |  |  | MAM-3 | -24.86 | 30.6 ± 3.8 | 12.1 ± 1.6 |
|  |  |  |  |  |  |  |  |  |  | MAM-4 | -24.659 | 26.7 ± 10.3 | 10.6 ± 4.1 |
| SDP23-4 | 280 | U4 – F8 | 180-250 | 3.47 ± 0.12 | 10 / 200 | 1 ± 1 | -0.777 | 1.549 | 0.774 | CAM | -3.413 | 55.7 ± 2.0 | **16.0 ± 0.9*** |
|  |  |  |  |  |  |  |  |  |  | MAM-3 | -3.461 | 49.0 ± 10.4 | 14.1 ± 3.0 |
|  |  |  |  |  |  |  |  |  |  | MAM-4 | -2.73 | 26.8 ± 11.5 | 7.7 ± 3.3 |
| SDP23-6 | 360 | U3 – F7 | 90-125 | 2.72 ± 0.11 | 107 / 5100 | 32 ± 4 | 0.004 | 0.473 | 0.236 | CAM | -59.735 | 56.5 ± 2.3 | **20.8 ± 1.2** |
|  |  |  |  |  |  |  |  |  |  | MAM-3 | -59.391 | 43.3 ± 5.4 | 15.9 ± 2.1 |
|  |  |  |  |  |  |  |  |  |  | MAM-4 | -58.848 | 47.4 ± 6.3 | 17.4 ± 2.5 |
| SDP23-7 | 480 | U3 – F5 | 90-125 | 2.71 ± 0.11 | 153 / 1900 | 31 ± 3 | -0.146 | 0.396 | 0.198 | CAM | -81.869 | 60.2 ± 2.0 | **22.2 ± 1.2*** |
|  |  |  |  |  |  |  |  |  |  | MAM-3 | -81.702 | 45.6 ± 5.5 | 16.8 ± 2.2 |
|  |  |  |  |  |  |  |  |  |  | MAM-4 | -81.662 | 44.0 ± 10.4 | 16.2 ± 3.9 |
| SDP23-3 | 620 | U2 – F4 | 212-250 | 2.12 ± 0.08 | 239 / 2400 | 30 ± 2 | 0.002 | 0.316 | 0.158 | CAM | -114.908 | 127.9 ± 3.3 | **60.4 ± 3.1*** |
|  |  |  |  |  |  |  |  |  |  | MAM-3 | -114.325 | 125.5 ± 12.6 | 54.1 ± 5.9 |
|  |  |  |  |  |  |  |  |  |  | MAM-4 | -114.291 | 134.2 ± 6.0 | 56.7 ± 6.7 |
| SDP23-2 | 670 | U1 – F3 | 90-125 | 2.08 ± 0.08 | 21 / 300 | 25 ± 6 | -0.150 | 1.069 | 0.534 | CAM | -6.253 | 257.0 ± 18.5 | **123.6 ± 10.4*** |
|  |  |  |  |  |  |  |  |  |  | MAM-3 | -6.305 | 218.3 ± 42.3 | 105.0 ± 20.9 |
|  |  |  |  |  |  |  |  |  |  | MAM-4 | -6.09 | 210.9 ± 52.7 | 101.3 ± 25.7 |

^a^ Weighted skewness scores have been calculated on log-transformed D_e_ values using Eq. 7-8 [41]. Critical skewness scores have been calculated using Eq. 16 . Critical skewness values are taken to be equivalent to twice the standard error of skewness score (95% C.I.) for single-grain D_e_ datasets, following the results of sensitivity analyses performed by Bailey and Arnold [54] and Arnold [55].

^b^ CAM = central age model; MAM-3 = 3-parameter minimum age model; MAM-4 = 4-parameter minimum age model [40].

^c^ MAM D_e_ estimates have been calculated after adding, in quadrature, a relative error of 20% to each individual D_e_ measurement error to approximate the underlying dose overdispersion observed in ‘ideal’ (well-bleached and unmixed) sedimentary samples [41, 54, 56, 57].

^d^ Maximum log likelihood score of the CAM, MAM-3 or MAM-4 fit. For a given sample, the *L_max_* score of the MAM-3 is expected to be substantially higher (i.e., at least 1.92 greater) than that of the CAM when the addition of the extra model parameter improves the fit to the data. Likewise, the *L_max_* score of the MAM-4 is expected to be significantly greater than that of the MAM-3 (by at least 1.92 when compared with the 95% C.I. of a *X*^2^ distribution) when the addition of the extra model parameter improves the fit to the data. If the extra parameter of the MAM-3 (or MAM-4) is not supported by the data, then its *L_max_* score will be similar to (i.e., within 1.92 of) the CAM (or MAM-3) *L_max_* score, indicating that the simpler age model explains the data equally well [41].

^e^ Mean ± total uncertainty (68% confidence interval), calculated as the quadratic sum of the random and systematic uncertainties. Total uncertainty on the final age estimate includes a systematic component of ±2% associated with laboratory beta-source calibration.

^f^ The preferred age for each sample is indicated with an asterisk. For these samples, the preferred age has been derived using the statistical age model that yielded the optimum *L_max_* score, following the criterion outlined in footnote ^d^ and Arnold [41].

**Table S8.** D_e_ summary statistics, age model results, and corresponding single-grain OSL ages for Salto de Piedra (Olavarría, Buenos Aires, Argentina). The total dose rates shown in this table have been calculated using a combination of high-resolution gamma-ray spectrometry and low-level beta counting measurements. The preferred age for each sample is shown in bold and indicated with an asterisk.

| **Sample** | **Sample**  **depth**  **(cm)** | **Level** | **Grain**  **size**  **(μm)** | **Total**  **dose rate**  **(Gy/ka)** | **Accepted/Measured** | **Overdis-persion (%)** | **Weighted skewness ^a^** | **Critical skewness**  **(95% C.I.)  ^a^** | **Critical skewness**  **(68% C.I.)  ^a^** | **Age Model**  **^b, c^** | ***L_max_* score ^d^** | **D_e_ (Gy) ^e^** | **Age (ka) ^e, f^** |
| --- | --- | --- | --- | --- | --- | --- | --- | --- | --- | --- | --- | --- | --- |
| SDP23-2 | 670 | U1 – F3 | 125-250 | 2.02 ± 0.08 | 17 / 800 | 47 ± 12 | -0.732 | 1.188 | 0.594 | CAM | -14.348 | 596.4 ± 84.9 | **295.8 ± 44.2*** |
|  |  |  |  |  |  |  |  |  |  | MAM-3 | -15.766 | 316.7 ± 74.9 | 157.1 ± 37.8 |
|  |  |  |  |  |  |  |  |  |  | MAM-4 | -12.052 | 288.3 ± 59.5 | 143.0 ± 30.2 |
| SDP23-1 | 700 | U1 – F3 | 90-125 | 2.30 ± 0.09 | 90 / 2400 | 47 ± 5 | -0.296 | 0.516 | 0.258 | CAM | -76.394 | 594.3 ± 35.8 | **257.4 ± 19.5*** |
|  |  |  |  |  |  |  |  |  |  | MAM-3 | -78.648 | 342.7 ± 41.4 | 148.5 ± 19.2 |
|  |  |  |  |  |  |  |  |  |  | MAM-4 | -74.511 | 318.3 ± 38.2 | 137.9 ± 17.7 |

^a^ Weighted skewness scores have been calculated on log-transformed D_e_ values using Eq. 7-8 [41]. Critical skewness scores have been calculated using Eq. 16 of Bailey and Arnold [54]. Critical skewness values are taken to be equivalent to twice the standard error of skewness score (95% C.I.) for single-grain D_e_ datasets, following the results of sensitivity analyses performed by Arnold [41].

^b^ CAM = central age model; MAM-3 = 3-parameter minimum age model; MAM-4 = 4-parameter minimum age model [40].

^c^ MAM D_e_ estimates have been calculated after adding, in quadrature, a relative error of 20% to each individual D_e_ measurement error to approximate the underlying dose overdispersion observed in ‘ideal’ (well-bleached and unmixed) sedimentary samples elsewhere [44, 56, 57].

^d^ Maximum log likelihood score of the CAM, MAM-3 or MAM-4 fit. For a given sample, the *L_max_* score of the MAM-3 is expected to be substantially higher (i.e., at least 1.92 greater) than that of the CAM when the addition of the extra model parameter improves the fit to the data. Likewise, the *L_max_* score of the MAM-4 is expected to be significantly greater than that of the MAM-3 (by at least 1.92 when compared with the 95% C.I. of a *X*^2^ distribution) when the addition of the extra model parameter improves the fit to the data. If the extra parameter of the MAM-3 (or MAM-4) is not supported by the data, then its *L_max_* score will be similar to (i.e., within 1.92 of) the CAM (or MAM-3) *L_max_* score, indicating that the simpler age model explains the data equally well [41].

^e^ Mean ± total uncertainty (68% confidence interval), calculated as the quadratic sum of the random and systematic uncertainties. Total uncertainty on the final age estimate includes a systematic component of ±2% associated with laboratory beta-source calibration.

^f^ The preferred age for each sample is indicated with an asterisk. For these samples, the preferred age has been derived using the statistical age model that yielded the optimum *L_max_* score, following the criterion outlined in footnote ^d^ and Arnold [41].

**Table S9.** D_e_ summary statistics, age model results, and corresponding single-grain TT-OSL ages for Salto de Piedra (Olavarría, Buenos Aires, Argentina). The total dose rates shown in this table have been calculated using a combination of high-resolution gamma-ray spectrometry and low-level beta counting measurements. The preferred age for each sample is shown in bold and indicated with an asterisk.

| Sample | Sample  depth  (cm) | Level | Grain  size  (μm) | Total  dose rate  (Gy/ka) | Accepted/Measured | Overdis-persion (%) | Weighted skewness ^a^ | Critical skewness  (95% C.I.)  ^a^ | Critical skewness  (68% C.I.)  ^a^ | Age Model  ^b, c^ | *L_max_* score ^d^ | D_e_ (Gy) ^e^ | Age (ka) ^e, f^ | Fading-corrected Age (ka) ^g^ |
| --- | --- | --- | --- | --- | --- | --- | --- | --- | --- | --- | --- | --- | --- | --- |
| SDP23-2 | 670 | U1 – F3 | 90-125 | 2.57 ± 0.08 | 16 / 16 | 20 ± 4 | 0.744 | 1.224 | 0.612 | CAM | 3.596 | 576.5 ± 29.7 | 224.6 ± 14.4* | 269.5 ± 13.0* |
|  |  |  |  |  |  |  |  |  |  | MAM-3 | 3.274 | 445.0 ± 22.3 | 173.4 ± 10.9 |  |
|  |  |  |  |  |  |  |  |  |  | MAM-4 | 3.807 | 415.7 ± 35.2 | 162.0 ± 15.1 |  |
| SDP23-1 | 700 | U1 – F3 | 90-125 | 2.77 ± 0.10 | 12 / 12 | 32 ± 7 | 0.061 | 1.414 | 0.707 | CAM | -3.56 | 364.1 ± 34.2 | 131.6 ± 13.5* | 185.1 ± 17.5* |
|  |  |  |  |  |  |  |  |  |  | MAM-3 | -2.903 | 236.3 ± 128.0 | 85.4 ± 46.4 |  |
|  |  |  |  |  |  |  |  |  |  | MAM-4 | -2.372 | 252.9 ± 26.2 | 91.4 ± 10.2 |  |

^a^ Weighted skewness scores have been calculated on log-transformed D_e_ values using Eq. 7-8 [41]. Critical skewness scores have been calculated using Eq. 16 of Bailey and Arnold [54]. Critical skewness values are taken to be equivalent to the standard error of skewness score (68% C.I.) for multi-grain D_e_ datasets, following the results of sensitivity analyses performed by Bailey and Arnold [54] and Arnold [55].

^b^ CAM = central age model; MAM-3 = 3-parameter minimum age model; MAM-4 = 4-parameter minimum age model [40].

^c^ MAM D_e_ estimates have been calculated after adding, in quadrature, a relative error of 5% to each individual D_e_ measurement error to approximate the underlying dose overdispersion observed in ‘ideal’ (well-bleached and unmixed) sedimentary samples [55].

^d^ Maximum log likelihood score of the CAM, MAM-3 or MAM-4 fit. For a given sample, the *L_max_* score of the MAM-3 is expected to be substantially higher (i.e., at least 1.92 greater) than that of the CAM when the addition of the extra model parameter improves the fit to the data. Likewise, the *L_max_* score of the MAM-4 is expected to be significantly greater than that of the MAM-3 (by at least 1.92 when compared with the 95% C.I. of a *X*^2^ distribution) when the addition of the extra model parameter improves the fit to the data. If the extra parameter of the MAM-3 (or MAM-4) is not supported by the data, then its *L_max_* score will be similar to (i.e., within 1.92 of) the CAM (or MAM-3) *L_max_* score, indicating that the simpler age model explains the data equally well [41].

^e^ Mean ± total uncertainty (68% confidence interval), calculated as the quadratic sum of the random and systematic uncertainties. Total uncertainty on the final age estimate includes a systematic component of ±2% associated with laboratory beta-source calibration.

^f^ The preferred age for each sample is indicated with an asterisk. For these samples, the preferred age has been derived using the statistical age model that yielded the optimum *L_max_* score, following the criterion outlined in footnote ^d^ and Arnold [41].

^g^ Fading correction undertaken using the procedure described in Huntley and Lamothe [27].

**Table S10.** D_e_ summary statistics, age model results, and corresponding pIR-IRSL_225_ ages for Salto de Piedra (Olavarría, Buenos Aires, Argentina). The total dose rates shown in this table have been calculated using a combination of high-resolution gamma-ray spectrometry and low-level beta counting measurements. The preferred age for each sample is shown in bold and indicated with an asterisk.

**SI References**

1. Prieto AR. Vegetational history of the Late glacial–Holocene transition in the grasslands of eastern Argentina. Palaeogeogr, Palaeoclim, Palaeoecol. 2000;157:167–88. <https://doi.org/10.1016/s0031-0182(99)00163-7>.

2. Morrone JJ. Biogeographical regionalisation of the Neotropical region. Zootaxa. 2014;3782:1–110. <https://doi.org/10.11646/zootaxa.3782.1.1>.

3. Tonni EP, Cione AL, Figini AJ. Predominance of arid climates indicated by mammals in the pampas of Argentina during the Late Pleistocene and Holocene. Palaeogeogr Palaeoclim Palaeoecol. 1999;147:257–81. <https://doi.org/10.1016/s0031-0182(98)00140-0>.

4. Sanz-Pérez D, Fernández MH, Tomassini RL, Montalvo CI, Beilinson E, Gasparini GM, et al. The Pampean region (Argentina) underwent larger variation in aridity than in temperature during the late Pleistocene: New evidence from the isotopic analysis of mammalian taxa. Quat Sci Rev. 2022;286:107555. <https://doi.org/10.1016/j.quascirev.2022.107555>.

5. Iriondo MH, Garcia NO. Climatic variations in the Argentine plains during the last 18,000 years. Palaeogeogr, Palaeoclim, Palaeoecol. 1993;101:209–20. <https://doi.org/10.1016/0031-0182(93)90013-9>.

6. Politis GG, Messineo PG, Stafford TW, Lindsey EL. Campo Laborde: A Late Pleistocene giant ground sloth kill and butchering site in the Pampas. Sci Adv. 2019;5:eaau4546. <https://doi.org/10.1126/sciadv.aau4546>.

7. Politis GG, Gutierrez MA, Scabuzzo C. Estado actual de las investigaciones en el sitio Arroyo Seco 2 (Partido de Tres Arroyos, provincia de Buenos Aires, Argentina). Universidad Nacional del Centro de la Provincia de Buenos Aires; 2014.

8. Salemme 31. M., Martínez G, Gutiérrez MA. Hunter-gatherers and Extinct Fauna during the Late Pleistocene/Holocene Transition in the Pampas Region (Argentina): An Overview. In: Gasparini GM, Rabassa J, editors. Late Cenozoic Fossil Mammals of Southern Cone of South America. Springer Nature; 2025.

9. Martínez G, Gutiérrez M. Paso Otero 5: a summary of the interdisciplinary lines of evidence for reconstructing early human occupation and paleoenvironment in the Pampean region, Argentina. In: Naturrelle MN d´Histoire, editor. Peuplement et Préhistoire de l´Ameriqué. 2011. p. 271–86.

10. Favier-Dubois CM, Villegas DH, Bonini R, Gómez GN, Steffan P, Bax M, et al. Alluvial deposit stratigraphy from the Salto de Piedra Paleontological Locality (Argentine Pampas): evidence from last glacial-interglacial cycles. Neues Jahrbuch Für Geologie Und Paläontologie - Abhandlungen. 2021;299:29–48. <https://doi.org/10.1127/njgpa/2021/0953>.

11. Christiansen P, Harris JM. Body size of Smilodon (Mammalia: Felidae). J Morphol. 2005;266:369–84. <https://doi.org/10.1002/jmor.10384>.

12. Manzuetti A, Perea D, Jones W, Ubilla M, Rinderknecht A. An extremely large saber-tooth cat skull from Uruguay (late Pleistocene–early Holocene, Dolores Formation): body size and paleobiological implications. Alcheringa: Australas J Palaeontol. 2020;44:332–9. <https://doi.org/10.1080/03115518.2019.1701080>.

13. Prevosti FJ, Forasiepi AM. Evolution of South American Mammalian Predators During the Cenozoic: Paleobiogeographic and Paleoenvironmental Contingencies. Springer Geol. 2018. <https://doi.org/10.1007/978-3-319-03701-1>.

14. Aitken MJ. Introduction to optical dating: the dating of Quaternary sediments by the use of photon-stimulated luminescence. Oxford University Press; 1998.

15. Murray AS, Wintle AG. Luminescence dating of quartz using an improved single-aliquot regenerative-dose protocol. Radiat Meas. 2000;32:57–73. <https://doi.org/10.1016/s1350-4487(99)00253-x>.

16. Buylaert J, Jain M, Murray AS, Thomsen KJ, Thiel C, Sohbati R. A robust feldspar luminescence dating method for Middle and Late Pleistocene sediments. Boreas. 2012;41:435–51. <https://doi.org/10.1111/j.1502-3885.2012.00248.x>.

17. Stevens T, Buylaert J-P, Murray AS. Towards development of a broadly-applicable SAR TT-OSL dating protocol for quartz. Radiat Meas. 2009;44:639–45. <https://doi.org/10.1016/j.radmeas.2009.02.015>.

18. Duval M, Guilarte V, Campaña I, Arnold LJ, Miguens L, Iglesias J, et al. Quantifying hydrofluoric acid etching of quartz and feldspar coarse grains based on weight loss estimates: implication for ESR and luminescence dating studies. Anc TL. 2018;36:1–14. <https://doi.org/10.26034/la.atl.2018.522>.

19. Leslie C. Analysing environmental radioactivity in soils and sediments using high-purity germanium gamma detectors at CSIRO Land and Water: Procedures and Protocols. Citeseer; 2009.

20. Guérin G, Mercier N, Adamiec G. Dose-rate conversion factors: update. Ancient TL. 2011;29:5–8.

21. Mejdahl V. Thermoluminescence dating: beta-dose attenuation in quartz grains. Archaeometry. 1979;21:61–72.

22. Brennan BJ. Beta doses to spherical grains. Radiat Meas. 2003;37:299–303. <https://doi.org/10.1016/s1350-4487(03)00011-8>.

23. Aitken M. Thermoluminescence Dating. London: Academic Press; 1985.

24. Readhead ML. Thermoluminescence dose rate data and dating equations for the case of disequilibrium in the decay series. Int J Radiat Appl Instrum Part D Nucl Tracks Radiat Meas. 1987;13:197–207. <https://doi.org/10.1016/1359-0189(87)90037-9>.

25. Prescott JR, Hutton JT. Cosmic ray contributions to dose rates for luminescence and ESR dating: Large depths and long-term time variations. Radiat Meas. 1994;23:497–500. <https://doi.org/10.1016/1350-4487(94)90086-8>.

26. Huntley DJ, Baril M. The K content of the K-feldspars being measured in optical dating or in thermoluminescence dating. Ancient TL. 1997;15:11–3.

27. Huntley DJ, Lamothe M. Ubiquity of anomalous fading in K-feldspars and the measurement and correction for it in optical dating. Canadian Journal of Earth Sciences. 2001;38:1093–106. <https://doi.org/10.1139/e01-013>.

28. Olley JM, Roberts RG, Murray AS. Disequilibria in the uranium decay series in sedimentary deposits at Allen’s cave, nullarbor plain, Australia: Implications for dose rate determinations. Radiat Meas. 1997;27:433–43. <https://doi.org/10.1016/s1350-4487(96)00114-x>.

29. Olley JM, Murray A, Roberts RG. The effects of disequilibria in the uranium and thorium decay chains on burial dose rates in fluvial sediments. Quat Sci Rev. 1996;15:751–60. <https://doi.org/10.1016/0277-3791(96)00026-1>.

30. Preusser F, Degering D. Luminescence dating of the Niederweningen mammoth site, Switzerland. Quat Int. 2007;164:106–12. <https://doi.org/10.1016/j.quaint.2006.12.002>.

31. Duller GAT. Distinguishing quartz and feldspar in single grain luminescence measurements. Radiat Meas. 2003;37:161–5. <https://doi.org/10.1016/s1350-4487(02)00170-1>.

32. Arnold LJ, Demuro M, Parés JM, Pérez-González A, Arsuaga JL, Castro JMB de, et al. Evaluating the suitability of extended-range luminescence dating techniques over early and Middle Pleistocene timescales: Published datasets and case studies from Atapuerca, Spain. Quat Int. 2015;389:167–90. <https://doi.org/10.1016/j.quaint.2014.08.010>.

33. Galbraith R. note on the variance of a background-corrected OSL count. Anc TL. 2002;20:49–51. <https://doi.org/10.26034/la.atl.2002.348>.

34. Jacobs Z, Duller GAT, Wintle AG. Interpretation of single grain De distributions and calculation of De. Radiat Meas. 2006;41:264–77. <https://doi.org/10.1016/j.radmeas.2005.07.027>.

35. Duller GAT. Assessing the error on equivalent dose estimates derived from single aliquot regenerative dose measurements. Anc TL. 2007;25:15–24. <https://doi.org/10.26034/la.atl.2007.403>.

36. Prado JL, Duval M, Demuro M, Santos-Arévalo FJ, Alberdi MT, Tomassini RL, et al. Refining the chronology of Middle/Late Pleistocene fossil assemblages in the Argentine Pampas. Quat Sci Rev. 2024;344:108958. <https://doi.org/10.1016/j.quascirev.2024.108958>.

37. Demuro M, Arnold LJ, Aranburu A, Sala N, Arsuaga J-L. New bracketing luminescence ages constrain the Sima de los Huesos hominin fossils (Atapuerca, Spain) to MIS 12. J Hum Evol. 2019;131:76–95. <https://doi.org/10.1016/j.jhevol.2018.12.003>.

38. Auclair M, Lamothe M, Huot S. Measurement of anomalous fading for feldspar IRSL using SAR. Radiat Meas. 2003;37:487–92. <https://doi.org/10.1016/s1350-4487(03)00018-0>.

39. Ficcarelli G, Coltorti M, Moreno-Espinosa M, Pieruccini PL, Rook L, Torre D. A model for the Holocene extinction of the mammal megafauna in Ecuador. J S Am Earth Sci. 2003;15:835–45. <https://doi.org/10.1016/s0895-9811(02)00145-1>.

40. Galbraith RF, Roberts RG, Laslett GM, Yosshida H, Olley JM. Optical dating of single and multiple grains of quartz from jinmium rock shelter, northern Australia: Part I, experimental design and statistical models. Archaeometry. 1999;41:339–64. <https://doi.org/10.1111/j.1475-4754.1999.tb00987.x>.

41. Arnold LJ, Roberts RG, Galbraith RF, DeLong SB. A revised burial dose estimation procedure for optical dating of youngand modern-age sediments. Quat Geochronol. 2009;4:306–25. <https://doi.org/10.1016/j.quageo.2009.02.017>.

42. Huntley DJ, Hancock R, Ancient T. The Rb contents of the K-feldspar grains being measured in optical dating. Ancient TL. 2001;19:43–6.

43. Katz D, Friess M. Technical Note: 3D From Standard Digital Photography of Human Crania—A Preliminary Assessment. Am J Phys Anthr. 2014;154:152–8. <https://doi.org/10.1002/ajpa.22468>.

44. Bowler JM, Johnston H, Olley JM, Prescott JR, Roberts RG, Shawcross W, et al. New ages for human occupation and climatic change at Lake Mungo, Australia. Nature. 2003;421:837–40. <https://doi.org/10.1038/nature01383>.

45. Jacobs Z, Duller GAT, Wintle AG, Henshilwood CS. Extending the chronology of deposits at Blombos Cave, South Africa, back to 140ka using optical dating of single and multiple grains of quartz. J Hum Evol. 2006;51:255–73. <https://doi.org/10.1016/j.jhevol.2006.03.007>.

46. Pawley SM, Bailey RM, Rose J, Moorlock BSP, Hamblin RJO, Booth SJ, et al. Age limits on Middle Pleistocene glacial sediments from OSL dating, north Norfolk, UK. Quat Sci Rev. 2008;27:1363–77. <https://doi.org/10.1016/j.quascirev.2008.02.013>.

47. Lewis RJ, Tibby J, Arnold LJ, Barr C, Marshall J, McGregor G, et al. Insights into subtropical Australian aridity from Welsby Lagoon, north Stradbroke Island, over the past 80,000 years. Quat Sci Rev. 2020;234:106262. <https://doi.org/10.1016/j.quascirev.2020.106262>.

48. Rees-Jones J. Optical dating of young sediments using fine-grain quartz. Ancient TL. 1995;13:9–14.

49. Rees‐Jones J, Tite MS. Optical dating results for british archaeological sediments. Archaeometry. 1997;39:177–87. <https://doi.org/10.1111/j.1475-4754.1997.tb00797.x>.

50. Mejdahl V. Internal radioactivity in quartz and feldspar grains. Ancient Tl. 1987;5:10–7.

51. Huntley DJ, Clague JJ. Optical Dating of Tsunami-Laid Sands. Quat Res. 1996;46:127–40. <https://doi.org/10.1006/qres.1996.0053>.

52. Huntley D, Lian OB. Using optical dating to determine when a sediment was last exposed to sunlight. In: Canada GS of, Bulletin, editors. Holocene climate and environmental change in the Palliser Triangle: a geoscientific context for evaluation the impacts of climate change on the southern Canadian prairies. 1999. p. 211–22.

53. Alappat L, Tsukamoto S, Singh P, Srikanth D, Ramesh R, Frechen M. Chronology of Cauvery Delta Sediments from Shallow Subsurface Cores Using Elevated-Temperature Post-IR IRSL Dating of Feldspar. Geochronometria. 2010;37 1:37–47. <https://doi.org/10.2478/v10003-010-0025-1>.

54. Bailey RM, Arnold LJ. Statistical modelling of single grain quartz De distributions and an assessment of procedures for estimating burial dose. Quat Sci Rev. 2006;25:2475–502. <https://doi.org/10.1016/j.quascirev.2005.09.012>.

55. Arnold LJ, Bailey RM, Tucker GE. Statistical treatment of fluvial dose distributions from southern Colorado arroyo deposits. Quat Geochronol. 2007;2:162–7. <https://doi.org/10.1016/j.quageo.2006.05.003>.

56. Arnold LJ, Demuro M, Duval M, Grün R, Sanz M, Costa AM, et al. Single-grain luminescence and combined U-series/ESR dating of the early Upper Palaeolithic Lagar Velho Rock Shelter, Leiria, Portugal. Quat Geochronol. 2024;83:101572. <https://doi.org/10.1016/j.quageo.2024.101572>.

57. Arnold LJ, Demuro M, Spooner NA, Prideaux GJ, McDowell MC, Camens AB, et al. Single-grain TT-OSL bleaching characteristics: Insights from modern analogues and OSL dating comparisons. Quat Geochronol. 2019;49:45–51. <https://doi.org/10.1016/j.quageo.2018.01.004>.
